# Comparative biophysical characterization: A screening tool for acetylcholinesterase inhibitors

**DOI:** 10.1371/journal.pone.0215291

**Published:** 2019-05-31

**Authors:** Devashree N. Patil, Sushama A. Patil, Srinivas Sistla, Jyoti P. Jadhav

**Affiliations:** 1 Department of Biotechnology, Shivaji University, Kolhapur, MS, India; 2 Institute for Structural Biology, Drug Discovery and Development, Virginia Commonwealth University, Richmond, Virginia, United States; College of Agricultural Sciences, UNITED STATES

## Abstract

Among neurodegenerative diseases, Alzheimer’s disease (AD) is one of the most grievous disease. The oldest cholinergic hypothesis is used to elevate the level of cognitive impairment and acetylcholinesterase (AChE) comprises the major targeted enzyme in AD. Thus, acetylcholinesterase inhibitors (AChEI) constitutes the essential remedy for the treatment of AD. The study aims to evaluate the interactions between natural molecules and AChE by Surface Plasmon Resonance (SPR). The molecules like alkaloids, polyphenols and substrates of AChE have been considered for the study with a major emphasis on affinity and kinetics. To better understand the activity of small molecules, the investigation is supported by both experimental and theoretical approach such as fluorescence, Circular Dichroism (CD) and molecular docking studies. Amongst the screened ones tannic acid showed promising results compared with others. The methodology followed here have highlighted many molecules with a higher affinity towards AChE and these findings may take lead molecules generated in preclinical studies to treat neurodegenerative diseases. Additionally, we suggest a unique signature for the heterogeneous analyte model using competitive experiments for analyzing simultanous interactions of both the analytes.

## Introduction

Alzheimer’s disease is the global age-related neurodegenerative disease which depicts 50–75 percentage (%) of the population of dementia all over the globe [1]. It is associated with behavioral changes, cognitive dysfunction, progressive memory deterioration and difficulty in daily living [2]. AD is marked by depletion of cholinergic synapses in the hippocampus and neocortex, resulting in insufficiency of the neurotransmitter acetylcholine (ACh).

Oxidative stress and neuroinflammation are the two main scrutinized factors responsible for AD. In AD there is mainly hyperphosphorylated tau, neurofibrillary tangles and hoarding of beta-amyloid plaques [3]. According to amyloid cascade hypothesis pathogenesis of AD is due to the flocculation of Aβ peptide in the brain ultimately leading to the formation of senile plaques. Formed plaques are responsible for neuronal cell death and eventually dementia [4]. Several pharmacological strategies have been revealed in the past two decades for hampering the aggregation of Aβ as a potent therapy to treat AD.

Acetylcholinesterase (AChE) (EC 3.1.1.7) from *Electrophorus electricus* is an ellipsoid shape enzyme. The enzyme active site is positioned in the deep and narrow gorge that becomes larger in the bottom, where the catalytic site is located. The active site comprises of esteratic site containing catalytic and anionic site with another site referred as the peripheral anionic site [5]. Rapid AChE enzyme activity accelerates Aβ aggregation [6]. Thus acetylcholinesterase inhibitors (AChEI) could arrest Aβ plaques formation [1] these strategies includes non-cholinergic and cholinergic treatments. Among the cholinergic proposition, the earliest approved drugs were AChEI for the control of the disease [7]. AChE drugs mainly dopenzil, rivastigmine and galanthamine hydrobromide approved by FDA [8] for treatment of AD which improves perception by elevating neurotransmission of ACh at cholinergic synapse through catabolic inhibition of acetylcholine into acetate and choline [9].

Since 2001, Galantamine hydrobromide is applicable to treat mild to moderate type of AD by possessing rapid and full absorption with oral bioavailability [10]. It was isolated from *Leucojum* sp, *Narcissus* sp and *Galanthus* sp [11] which is used nowadays and clinically tested. Galanthamine is naturally available from many plant sources having promising AChE inhibitory activity, but there is still interest to search for non-alkaloid molecules for the inhibition of AChE and polyphenols have emerged as a promising alternatives [5]. Of the available drugs, substrate specificity, target binding and side effects were not studied in great detail.

In recent years there is much more significant interest in the mechanism of action for various polyphenols against neurodegenerative diseases. Especially in AD, polyphenols have shown the ability to address the etiology of neurological disorders as they deteriorate their complex physiology by regulating therapeutic targets [12] with reduced in the risk of AD with age [13,14]. Also, polyphenols can reduce inflammation by behaving as anti-inflammatory agents [15] and by decreasing the risk of oxidative stress [16]. There are reports available in the literature for various flavones, isoflavones, flavanols, anthocyanidins, curcuminoids and stilbenes for a useful role in inhibiting AChE enzyme. [1,17]. Also, various plant extracts rich in phytomolecules especially alkaloids having a capacity to inhibit AChE [2,18,19].

Several methods are accessible for the detection of AChEI, most of them dependent on photometric sensing of AChE activity. Other techniques like chemiluminescence, electrochemical detection, thin layer chromatography, microplate assay are routinely used to detect cholinesterase inhibitors [18]. Fast Blue B salt reagent and various spectroscopic methods [20] have also been addressed to study AChEI. There are very limited reports available regarding the screening of AChEI’s by using SPR. Recently, detection of AChEI has been reported with two inhibitors neostigmine and eserine [21] and the affinity of few drug molecules with AChE [22] by using SPR. However, screening and kinetic analysis followed by structure studies of different molecules using SPR for inhibition of AChE can be a fast and rapid alternative to identify potential drug molecules [23–26].

SPR biosensors have gained an important role in drug discovery. It is a real-time, label-free detection method used for studying binding affinity and kinetics of different biomolecules such as antigen-antibody, DNA-protein, receptor-analyte, enzyme-substrate and enzyme-inhibitor interactions [23]. This technique has also been used for screening of inhibitors with different enzymes such as tyrosinases where inhibition of tyrosinase was focused on preventing browning reactions in the food industry and cosmetics for fairness purpose [27]. In this present study, the interactions of different molecules with immobilized AChE enzyme has been analyzed using SPR biosensor. Competitive analyte binding study was performed to explore the interactions of two molecules simultaneously on the immobilized ligand on the gold sensor chip surface resulting in two sets of rate constants for each reaction. The structural changes of the AChE in the presence of inhibitors were analyzed using spectroscopic, fluorescence, CD and molecular docking studies.

The data generated in the study will be further helpful to screen several inhibitors and to grade the affinities of different molecules towards AChE enzyme. Very few reports describing the competitive binding studies are available in the literature; this work seems to be the first report studying the practical avidity concept. Such work will open a new era for the multidimensional inhibition and kinetic study of biomolecules.

## Materials and methods

### Chemicals and reagent

Sensor Chip Carboxy Methyl 5 (CM5), N-ethyl-N’-(dimethyl aminopropyl)-carbodiimide (EDC), N-hydroxysuccinimide (NHS), ethanolamine-HCl, phosphate buffer saline (PBS), surfactant P20, glycine HCl (hydrochloric acid) sampling vials and caps were obtained from GE Healthcare Life Sciences, Uppsala, Sweden. The molecules like galanthamine hydrobromide, lycorine hydrochloride, crocin, acetylthiocholine iodide (ATCI), 5,5^ꞌ^-dithiobis-(2-nitrobenzoic acid) (DTNB), carbachol, rutin hydrate, pyrogallol, quercetin, catechin hydrate and acetylcholinesterase from *Electrophorus electricus* (Electric eel) were purchased from Sigma (St. Louis, MO, USA) whereas, tannic acid, caffeine was obtained from Himedia, Mumbai, India. Galanthamine drug (Galamer 4) (Sun Pharmaceutical Industries Ltd, India) was purchased from a pharmacy shop from local market of Kolhapur, India. All chemicals in this study were of the highest purity and molecular purity grade. Milli Q (Millipore, Sigma, USA) water was used for preparing buffers and reagents.

### Enzyme activity assay

The assay was performed with some modifications of Ellman et al (1961) [28]. Concisely, the reaction mixture had a final volume of 2200 μl containing PBS (200 milli molar (mM), 137 mM NaCl, 2.7 mM KCl, pH 7.7) and enzyme (2 units ml^-1^), the reaction was started by adding 1mM DTNB and 7.5 mM ATCI and further incubated for 5 minutes (min) at 37°C (degree celsius). The hydrolysis was monitored by the formation of yellow color (5-thio-2-nitrobenzoate anion) and the absorbance was measured at 412 nm by using spectrophotometer (UV-1800, Shimadzu, Japan).

### Inhibition assay for acetylcholinesterase activity

The inhibitory activity assay was carried out with slight modifications [29]. AChE (2 unitsml^-1^) with PBS (200 mM, pH 7.7) was incubated with different molecules like galanthamine hydrobromide, galamer4, caffeine, lycorine hydrochloride, crocin, carbachol, rutin hydrate, pyrogallol, quercetin, catechin hydrate, tannic acid which were prior solubilized in PBS (200 mM, pH 7.7) for 10 min at 37°C. Then 1mM DTNB and 7.5 mM ATCI were added and finally, the reaction was monitored after 5 min and amount of yellow color in the mixture was determined by optical density at 412 nm using Shimadzu UV visible spectrophotometer. Galanthamine hydrobromide was used as a positive control. The inhibitory percentage of AChE was calculated as follows:

%inhibition = 100[A − B] − [C − D]/[A − B]

A is the OD without test substance, B is the OD without test substance and AChE, C is the OD with the test substance, D is the OD at 412 nm with the test substance and without AChE. The percentage of inhibition was expressed as the percentage required for 50% inhibition (IC_50_) of the enzyme.

#### Data analysis

The relative percentage inhibition were estimated in triplicates and values were expressed as mean ± SEM (n = 3).

### Surface Plasmon Resonance (SPR) studies

The protocol was followed with some modifications for enzyme immobilization, scouting and screening [27]. SPR interactions were performed using a Biacore X100 optical biosensor (GE Healthcare Life Sciences, Bangalore, India) at 25°C. SPR analysis was carried out in PBS (200 mM, pH 7.7) from the analyte stock solutions, working solutions were diluted before passing on to the sensor surface. Data collection was done with the help of Biacore control software ver 2.0.2 Sets of experiments were carried out by monitoring change in the refractive index as a function of time under constant flow conditions. The relative amount of inhibitor bound to the AChE was determined by measuring the net increase in the refractive index over time compared to buffer as a control. During each run, there is an inline subtraction of the reference surface. Change is reported regarding response units (RU). The surface was washed with 5 cycles of running buffer (PBS) before next analytes.

### Immobilization scouting

To determine suitable pH for immobilization, pH scouting was performed without any modification in the sensor chip surface. The enzyme solutions were prepared in acetate buffer ranging with pH 4.0–5.5 and were further injected over the chip surface before the activation of N-ethyl-N-(dimethylaminopropyl)-carbodiimide (EDC) and N-hydroxysuccinimide (NHS). PBS was used as a running buffer for this procedure.

### Enzyme immobilization

As per pH scouting studies, AChE enzyme (100μg) was dissolved in 100 mM sodium acetate buffer pH 4.5 and was immobilized to the CM5 sensor chip by using the amine coupling method. The flow rate was kept constant during immobilization at 10 μlmin^-1^, the surface of flow cell was activated by 1:1 mixture of 100 mM EDC and 100 mM NHS (both dissolved in water) for 7 min. AChE was injected for 7 min, and residual activated carboxymethyl groups were blocked on the sensor surface by 7 min injection of 1M ethanolamine, pH 8.5. In this particular study, flow cell 1 was blank immobilized (without protein) and used as a reference. Inline and double referencing was done for the buffer RU compensation.

### Screening of different molecules by using SPR

To study the interactions of different small phytomolecules like (crocin, rutin hydrate, pyrogallol, quercetin, catechin hydrate and tannic acid) among the alkaloids (galanthamine hydrobromide, galamer4, caffeine and lycorine hydrochloride) and ATCI (substrate) and carbachol (substrate analogue) were dissolved in 10 mM PBS containing 0.005% P20 and were injected on sensor chip.

Similarly, the interactions of heterogenous analytes like substrate-inhibitor (galanthamine hydrobromide and ATCI), (tannic acid and ATCI), (crocin and ATCI) and inhibitor-inhibitor (galanthamine hydrobromide and tannic acid) (galanthamine hydrobromide and crocin) with concentrations used before for individual small molecules were studied on the immobilized chip. Here the model describes this competitive situation and returns two sets of rate constants, one for each reaction taking into consideration two different analytes. The same buffer was used as running buffer. The constant flow rate was maintained throughout the kinetics (45 μlmin^-1^), contact time and dissociation time was kept at 120 second (s). 10 mM glycine pH 3.0 for the time period of 30 s was used for regeneration. The experiments were carried at different concentrations of inhibitors.

### Data analysis

The analysis of data was carried out with X100 evaluation software ver 2.0.2 and fitted to two state fit for individual molecules and for competitive experiments a heterogeneous analyte binding model.

### Circular dichroism studies

The far-UV CD region (190–260 nm) was obtained at room temperature and was analyzed on JASCO J-1500 (Jasco, MD, USA) spectropolarimeter at 25°C. Protein solutions 0.5 milligram per milliliter (mgml^-1^) was prepared in 10 mM PBS at pH 7.7. Scans were performed at room temperature using 0.1 cm path length quartz cuvette with 8 s differential integration time at a scan rate of 100 nano meter per minute (nm min^-1^). The CD spectrum of AChE enzyme individually and with different small molecules, also in combination was recorded. All spectra resulted from averaging three scans were corrected from the buffer blank. Results were expressed as molar ellipticity (°cm^2^ dmol^-1^) based on a mean amino acid residue weight (MRW) 110 Da for AChE. The molar ellipticity was determined as [θ obs] = {100 × (MRW) × θobs/*cl*}, where θ is the observed ellipticity in degrees at a given wavelength, *c* is the protein concentration in mgml^-1^, and l is the path length in cm [24].

### Data analysis

The relative percentages of the secondary structure elements were estimated in triplicates using Manager Suite software.

### Fluorescence spectral studies

The fluorescence intensities were recorded using 0.5 mgml^-1^ protein concentration with an excitation wavelength of 365 nm and an emission wavelength of 400–700 nm with slit widths of 20 nm using MY14410002 fluorescence spectrophotometer (Agilent Technologies, USA) at 25°C. Protein solutions of 0.5 mgml^-1^ were prepared in 10 mM PBS at pH 7.7. Scans were performed at room temperature using a 1 cm path length quartz cuvette with 1 s differential integration time at a scan rate of 600 nm min^-1^. The maximum emission wavelength λ_max_ for the AChE was 415 nm. The subsequent changes in fluorescence were measured at this wavelength. The protein solutions of 0.5 mgml^−1^ were incubated with and without galanthamine hydrobromide, galamer4, caffeine, lycorine hydrochloride, crocin, carbachol, cyanidin chloride, rutin hydrate, pyrogallol, quercetin, catechin hydrate, tannic acid and also in combination of two molecules like galanthamine hydrobromide and ATCI, tannic acid and ATCI, crocin and ATCI, galanthamine hydrobromide and tannic acid and finally galanthamine hydrobromide and crocin which were first dissolved in PBS at concentration showing 50% (IC_50_) inhibition with AChE for 10 min and were then used to obtain the fluroscence readings.

### Molecular modeling using SwissDock

Molecular modeling was done using Swiss Dock. This web-based service is based on the docking software EADock DSS [30]. This online service was selected as it has an interface to add in desired protein from Protein Data Bank; PDB and ligand structures from Zinc database, modify docking parameters and visualize most favorable docked complexes. Results of the Swiss Dock service were visualized by UCSF Chimera [31] and images obtained were presented. The lowest free binding energy prediction was used to explain the interactions. Crystal structure of human recombinant AChE (4EY7) was used in these docking studies. All the molecules were taken from Zinc database. UCSF Chimera 1.12 software was used for visualization of the results and creating images.

## Results

### Acetylcholinesterase inhibition assay and SPR analysis

**Fig 1:**
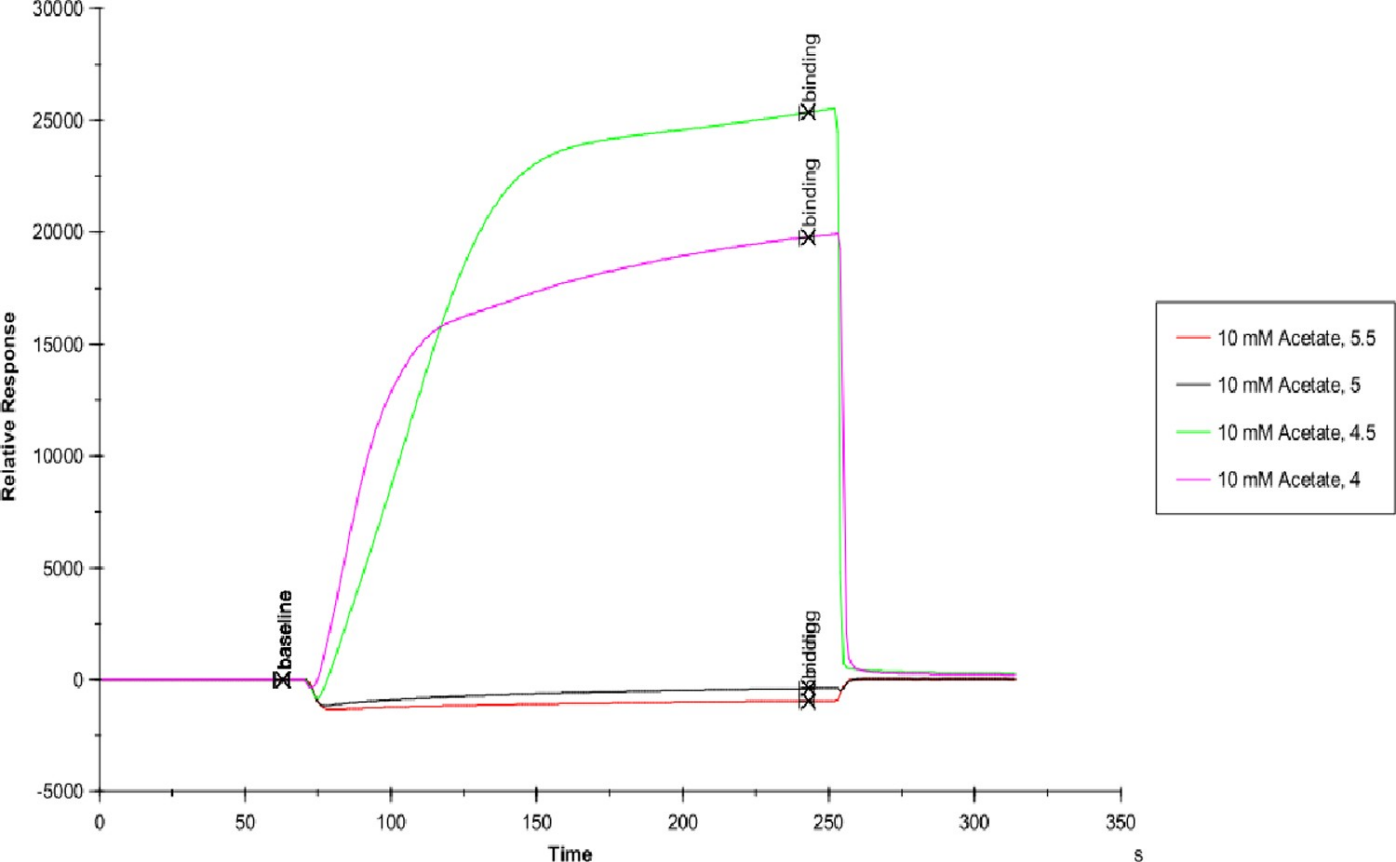
Immobilization scouting results on an unactivated biacore CM5 chip. Binding increases to the dextran matrix on chip surface as pH reduces from 5.5 to 4.5. The best immobilization pH for AChE is 4.5.

**Fig 2:**
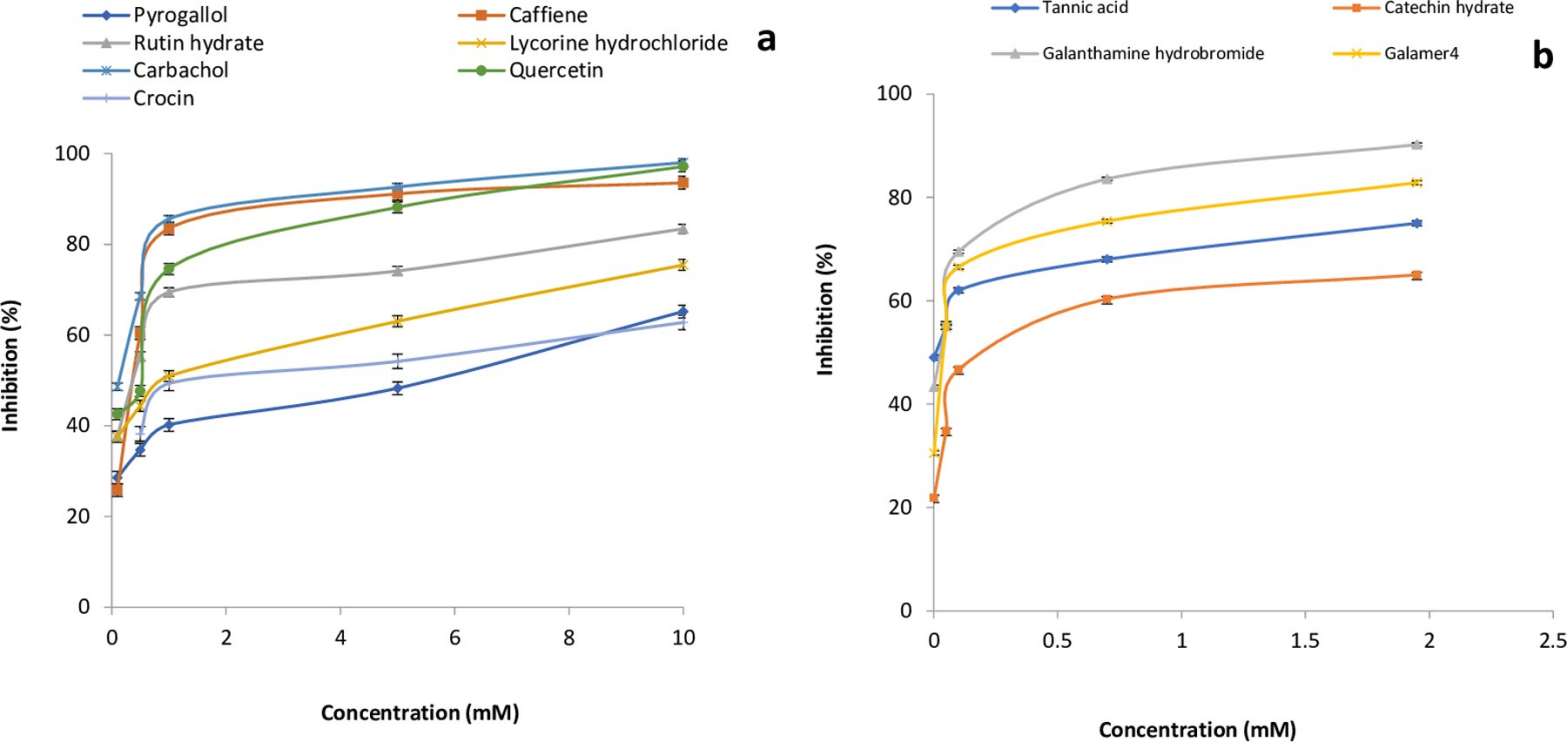
**a)** Effect of molecules- quercetin, rutin hydrate, lycorine hydrochloride, caffeine, carbachol, pyrogallol and crocin concentrations on the AChE activity using ATCI as substrate. The reaction mixture contained 7.5 mM of the substrate in a final concentration of 200 mM phosphate buffer saline (PBS), pH 7.7 at 37°C. **b)** Effect of molecules- tannic acid, catechin hydrate, galanthamine hydrobromide and galanthamine tablet concentrations on the AChE activity using ATCI as substrate. The reaction mixture contained 7.5 mM of the substrate in a final concentration of 200 mM phosphate buffer saline (PBS), pH 7.7 at 37°C.

**Fig 3:**
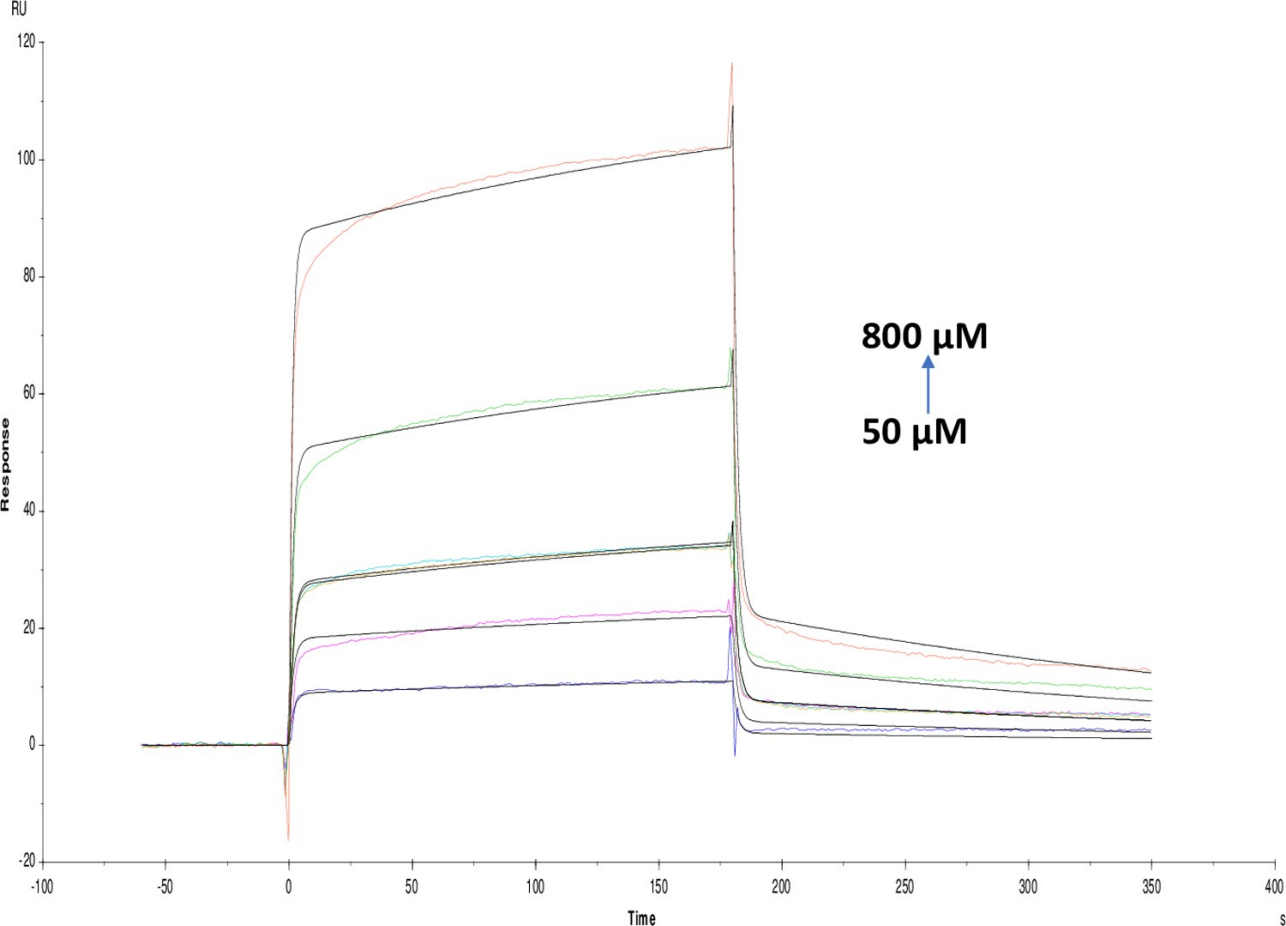
Binding sensorgram for galanthamine hydrobromide interaction with immobilized AChE at 25°C. Increasing concentrations of galanthamine hydrobromide from 50–800 micro molar (μM) were injected over the enzyme surface. The flow rate is maintained at 45 micro litre per minute (μl min^-1^). Contact time and dissociation time was kept at 120 s and 200 s. Regeneration was carried out using 10 mM glycine HCl with pH 3.0 for 30 s and at 30 μl min^-1^. The data analysis was done using Biacore X100 evaluation software ver 2.0.2 and data was fit to two state reaction. The resulting equilibrium dissociation constants KD, kinetic association k_on_ and dissociation k_off_ rates are given in Table 2.

**Fig 4:**
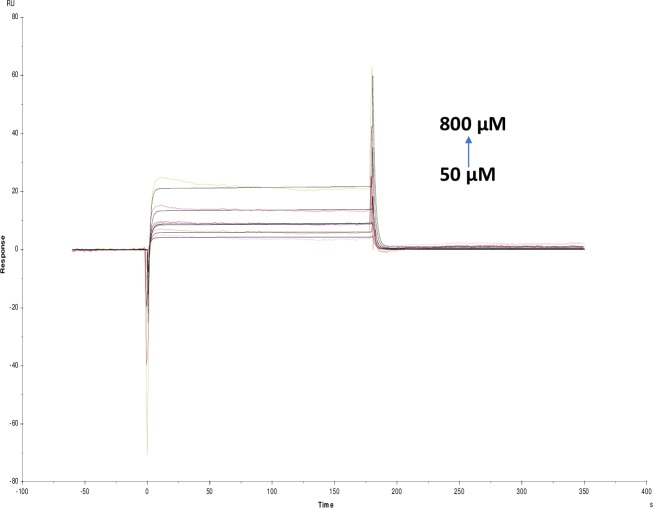
Binding sensorgram for galamer4 interaction with immobilized AChE. Increasing concentrations of galanthamine tablet from 50 μM-800 μM were injected over the enzyme surface at 25°C. Flow rate is maintained at 45 μl min^-1^. Contact time and dissociation time was kept at 120 s and 200 s. Regeneration was carried out using 10 mM glycine HCl with pH 3 for 30 s and at 30 μl min^-1^. The data analysis was done using Biacore X100 evaluation software ver 2.0.2 and data was fit to two state. The resulting equilibrium dissociation constants KD, kinetic association kon and dissociation koff rates are given in Table 2.

**Fig 5:**
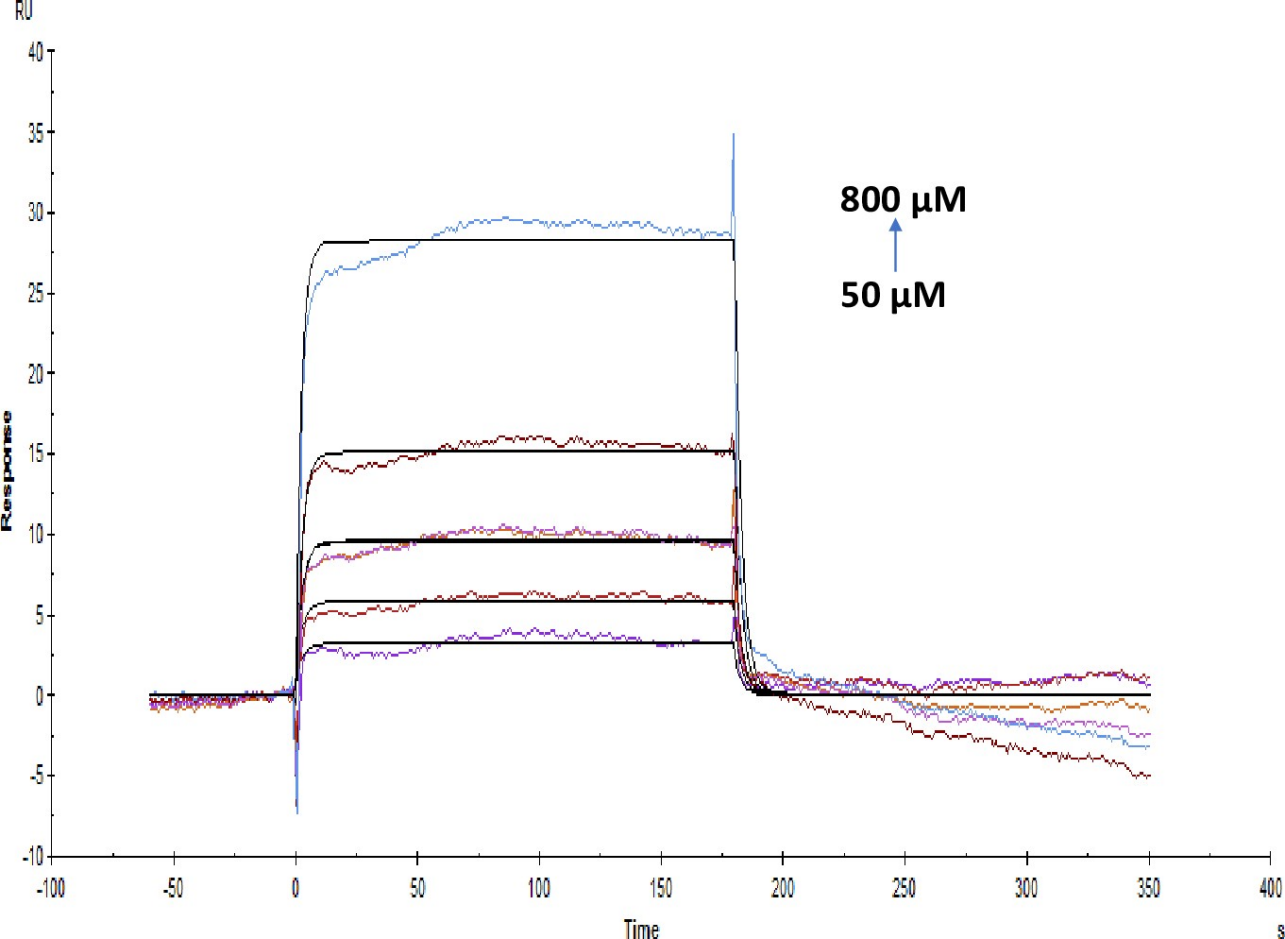
Binding sensorgram for lycorine hydrochloride interaction with immobilized AChE. Increasing concentrations of lycorine hydrochloride 50 μM-800 μM from were injected over enzyme surface. The flow rate is maintained at 45 μl min^-1^. Contact time and dissociation time was kept at 120 s and 200 s. Regeneration was carried out using 10 mM glycine HCl with pH 3 for 30 s and at 30 μl min^-1^. The data analysis was done using Biacore X100 evaluation software ver 2.0.2 and data was fit to two state. The resulting equilibrium dissociation constants KD, kinetic association kon and dissociation koff rates are given in Table 2.

**Fig 6:**
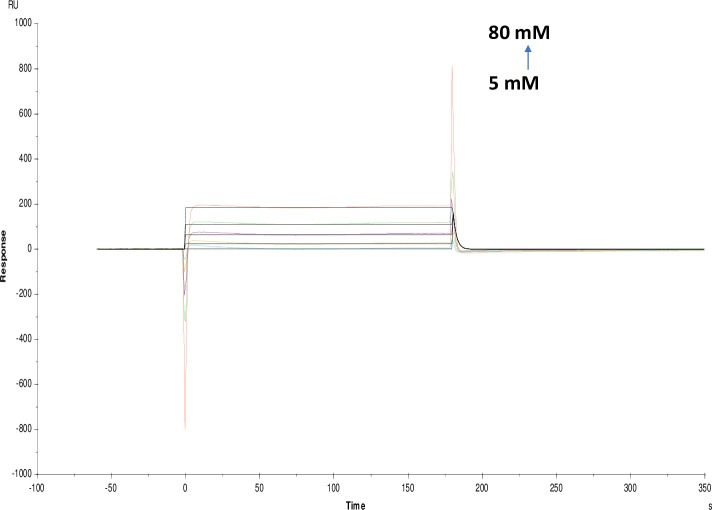
Binding sensorgram for caffeine interaction with immobilized AChE. Increasing concentrations of caffeine from 5 mM-80 mM were injected over enzyme surface at 25°C. The flow rate is maintained at 45 μl min^-1^. Contact time and dissociation time was kept at 120 s and 200 s. Regeneration was carried out using 10 mM glycine HCl with pH 3 for 30 s and at 30 μl min^-1^. The data analysis was done using Biacore X100 evaluation software ver 2.0.2 and data was fit to two state. The resulting equilibrium dissociation constants KD, kinetic association kon and dissociation koff rates are given in Table 2.

Affinity and kinetics of small molecules were studied on AChE enzyme using the SPR technique. The sensitivity required for detecting interaction with small molecules with weak affinities is dependent on the immobilization level, the size of the target molecule and the screening concentrations. Immobilization scouting results showed pH 4.5 (Fig 1) suitable for the immobilization of AChE on a sensor chip with 7732 Resonance Units (RU) at concentrations of 100 μgml^-1^. Some of the small molecules used in these studies were of alkaloid groups namely galanthamine hydrobromide, galamer4, caffeine and lycorine hydrochloride. SPR study revealed that the affinity of each alkaloid molecule towards AChE is different with KD values for galanthamine hydrobromide (1.039×10^-3^M) (Molar), galamer4 (1.753×10^-3^M), caffeine (1.738×10^-5^M) and lycorine hydrochloride (5.533×10^-4^M). These results were confirmed by enzyme inhibition studies where galanthamine hydrobromide and galamer4 showed the similar IC_50_ value of 0.001±0.001mM (Fig 2) and somewhat similar KD. The IC_50_ values of caffeine and lycorine hydrochloride are reported in the (Table 1). Among the alkaloids, caffeine showed a higher affinity towards AChE compared to galanthamine hydrobromide (Figs 3–6).

**Table 1:** IC_50_ values of different small molecules on AChE.

Sl. No.	Molecule name	IC_50_ in mM
1	Galanthamine hydrobromide	0.001±0.001
2	Galamer4	0.001±0.002
3	Caffeine	0.38±0.015
4	Lycorine hydrochloride	1.79±0.01
5	Carbachol	0.21±0.015
6	Quercetin	0.43±0.01
7	Rutin hydrate	1.9±0.09
8	Pyrogallol	3.8±0.1
9	Catechin hydrate	0.09±0.001
10	Tannic acid	0.027±0.002
11	Crocin	0.92±0.23

Values were expressed as mean ± SEM (n = 3).

**Fig 7:**
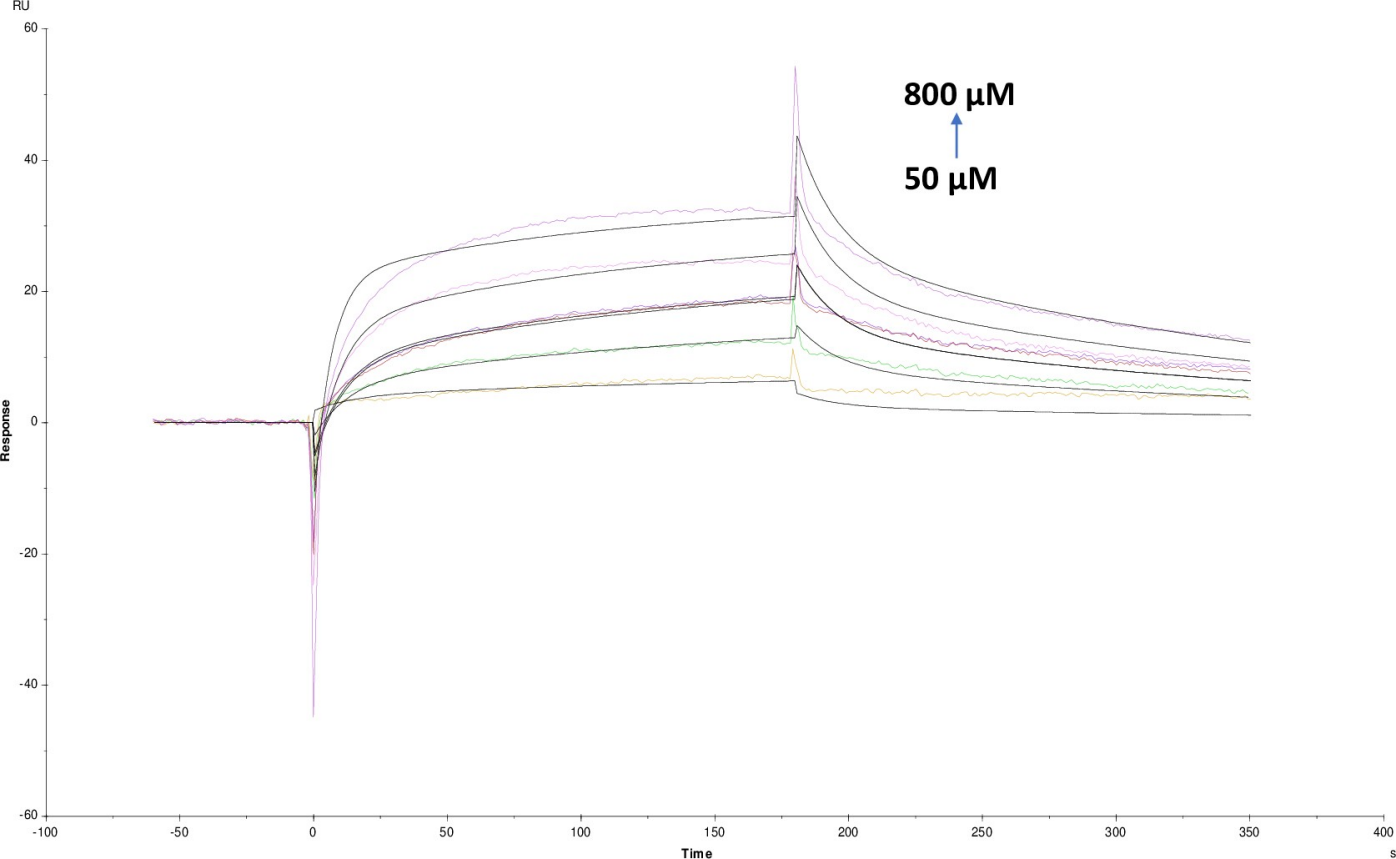
Binding sensorgram for quercetin interaction with immobilized AChE. Increasing concentrations of quercetin from 50 μM-800 μM were injected over the enzyme surface at 25°C. The flow rate is maintained at 45 μl min^-1^. Contact time and dissociation time was kept at 120 s and 200 s. Regeneration was carried out using 10 mM glycine HCl with pH 3 for 30 s and at 30 μl min^-1^. The data analysis was done using Biacore X100 evaluation software ver 2.0.2 and data was fit to two state. The resulting equilibrium dissociation constants KD, kinetic association kon and dissociation koff rates are given in Table 2.

**Fig 8:**
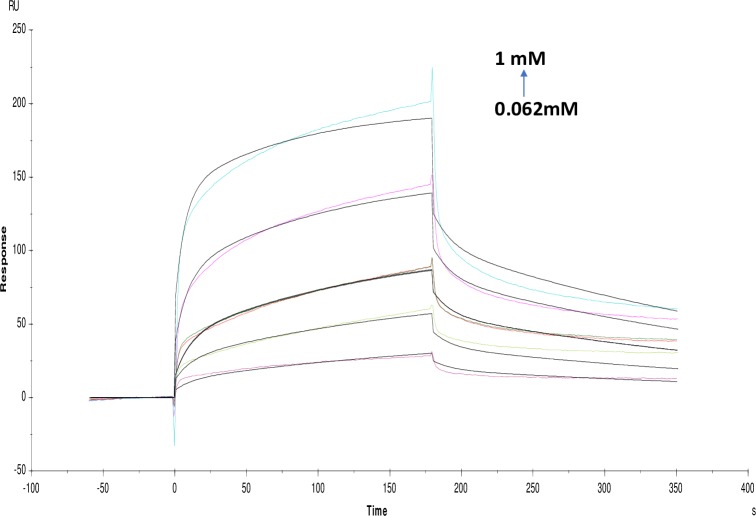
Binding sensorgram for catechin hydrate interaction with immobilized AChE. Increasing concentrations of catechin hydrate 0.062 mM-1 mM from were injected over the enzyme surface at 25°C. The flow rate is maintained at 45 μl min^-1^. Contact time and dissociation time was kept at 120 s and 200 s. Regeneration was carried out using 10 mM glycine HCl with pH 3 for 30 s and at 30 μl min^-1^. The data analysis was done using Biacore X100 evaluation software ver 2.0.2 and data was fit to two state. The resulting equilibrium dissociation constants KD, kinetic association kon and dissociation koff rates are given in Table 2.

**Fig 9:**
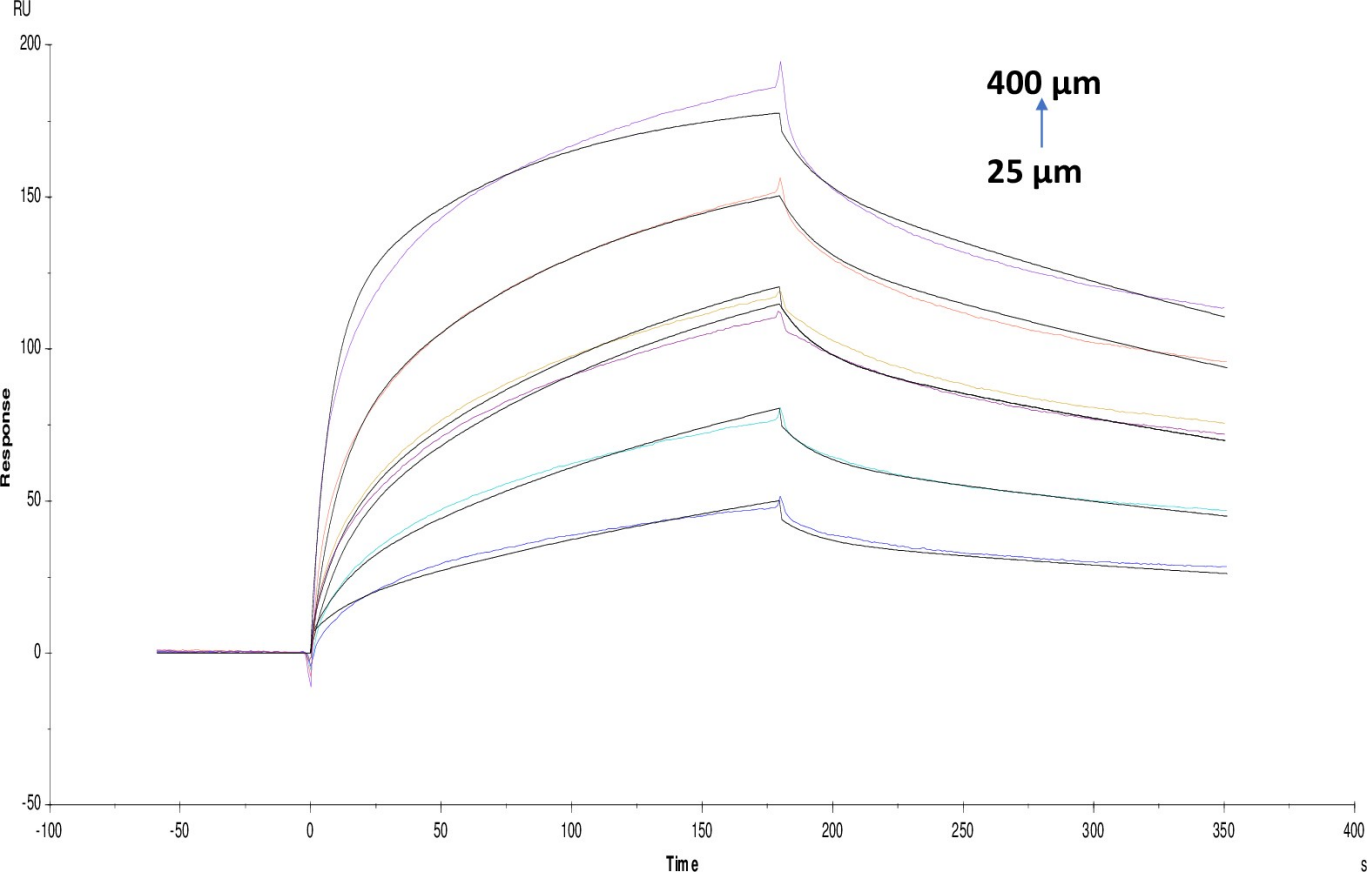
Binding sensorgram for pyrogallol interaction with immobilized AChE. Increasing concentrations of pyrogallol from 25 μM-400 μM were injected over the enzyme surface at 25°C. The flow rate is maintained at 45 μl min^-1^. Contact time and dissociation time was kept at 120 s and 200 s. Regeneration was carried out using 10 mM glycine HCl with pH 3 for 30 s and at 30 μl min^-1^. The data analysis was done using Biacore X100 evaluation software ver 2.0.2 and data was fit to two state. The resulting equilibrium dissociation constants KD, kinetic association kon and dissociation koff rates are given in Table 2.

**Fig 10:**
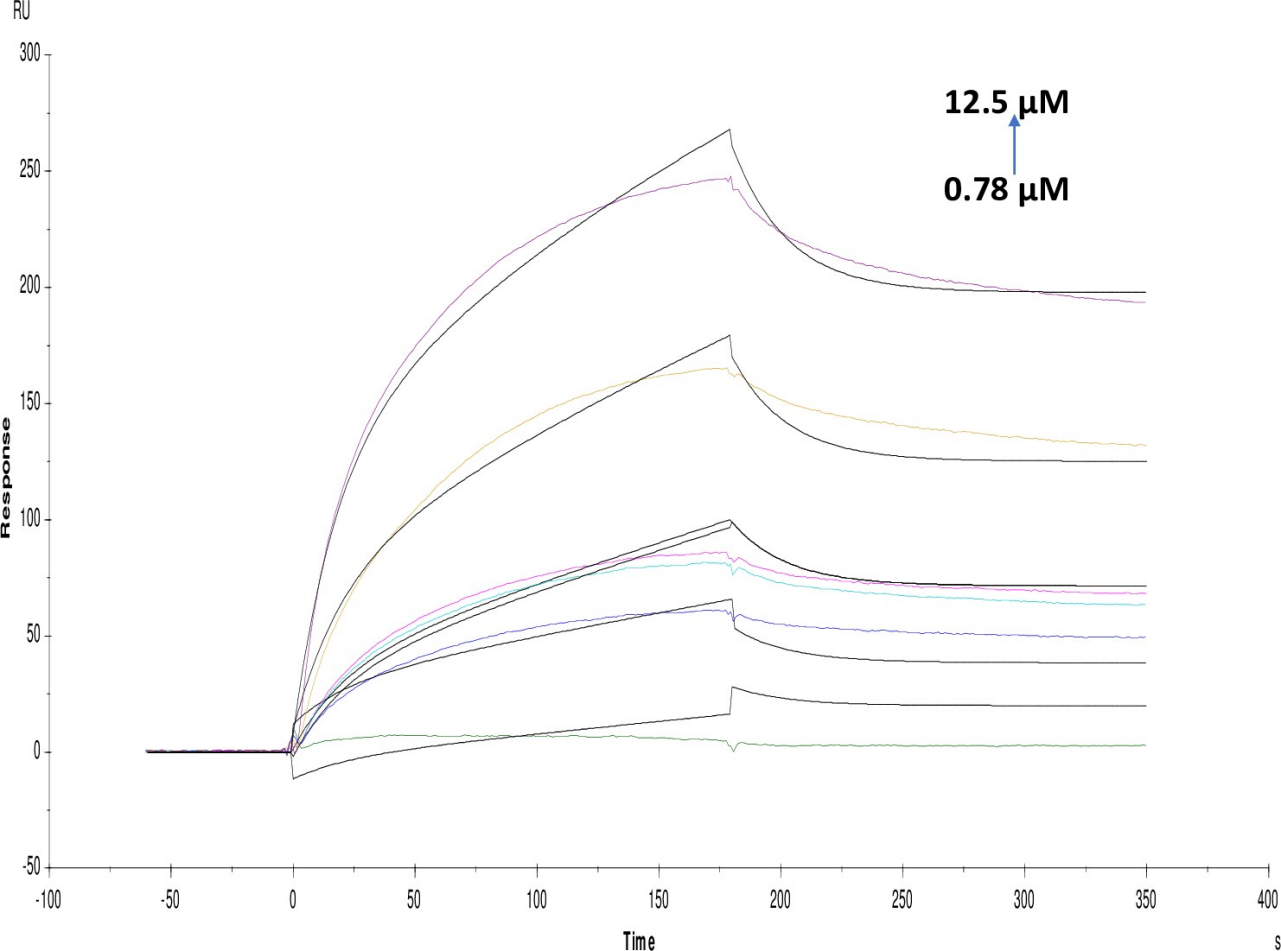
Binding sensorgram for tannic acid interaction with immobilized AChE. Increasing concentrations of tannic acid from 0.78 μM-12.5 μM were injected over the enzyme surface at 25°C. The flow rate is maintained at 45 μl min^-1^. Contact time and dissociation time was kept at 120 s and 200 s. Regeneration was carried out using 10 mM glycine HCl with pH 3 for 30 s and at 30 μl min^-1^. The data analysis was done using Biacore X100 evaluation software ver 2.0.2 and data was fit to two state. The resulting equilibrium dissociation constants KD, kinetic association kon and dissociation koff rates are given in Table 2.

**Fig 11:**
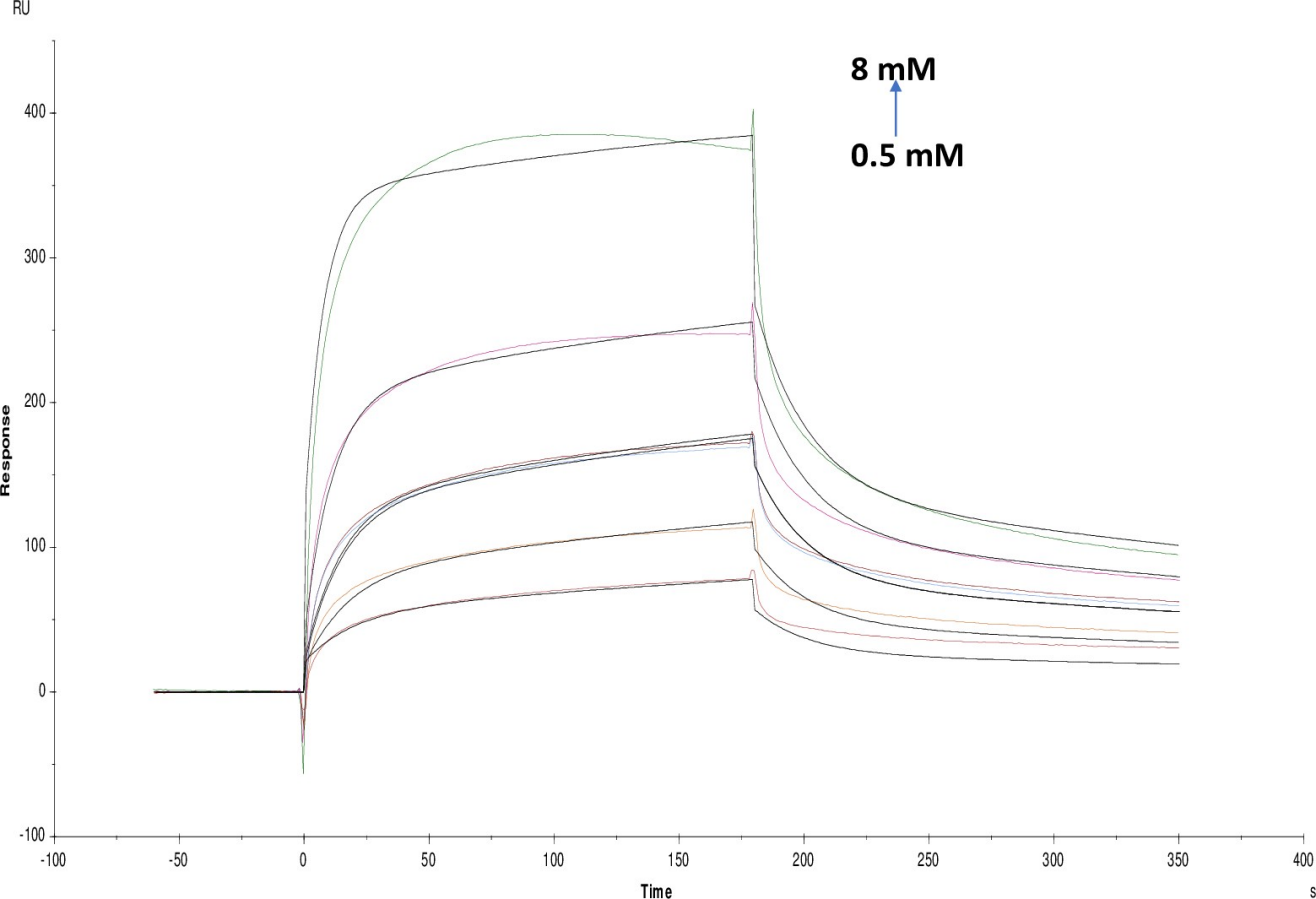
Binding sensorgram for rutin hydrate interaction with immobilized AChE. Increasing concentrations of rutin hydrate from 0.5 mM-8 mM were injected over enzyme surface at 25°C. The flow rate is maintained at 45 μl min^-1^. Contact time and dissociation time was kept at 120 s and 200 s. Regeneration was carried out using 10 mM glycine HCl with pH 3 for 30 s and at 30 μl min^-1^. The data analysis was done using Biacore X100 evaluation software ver 2.0.2 and data was fit to two state. The resulting equilibrium dissociation constants KD, kinetic association kon and dissociation koff rates are given in Table 2.

Many of the polyphenols reported earlier as potential AChE inhibitors which can be multipotent drugs against AD having less toxicity as compared with alkaloids [20]. Whereas, non-alkaloids in the present study showed different KD value for quercetin (2.122×10^-4^M), catechin hydrate (1.734×10^-4^M), pyrogallol (3.141×10^-5^M), tannic acid (2.250×10^-8^M) and rutin hydrate (1.184×10^-3^M) (Figs 7–11). Among them, tannic acid showed higher affinity with AChE followed by pyrogallol, quercetin and catechin hydrate. Moreover, molecules like quercetin, isoquercetin, rutin, genistein, kaempferol and catechin were studied for acetylcholinesterase inhibition activity [32]. This study has been supported and further characterized by the biophysical, structural and spectroscopic studies. The IC_50_ values of these non-alkaloid molecules were presented in the given (Table 1).

**Fig 12:**
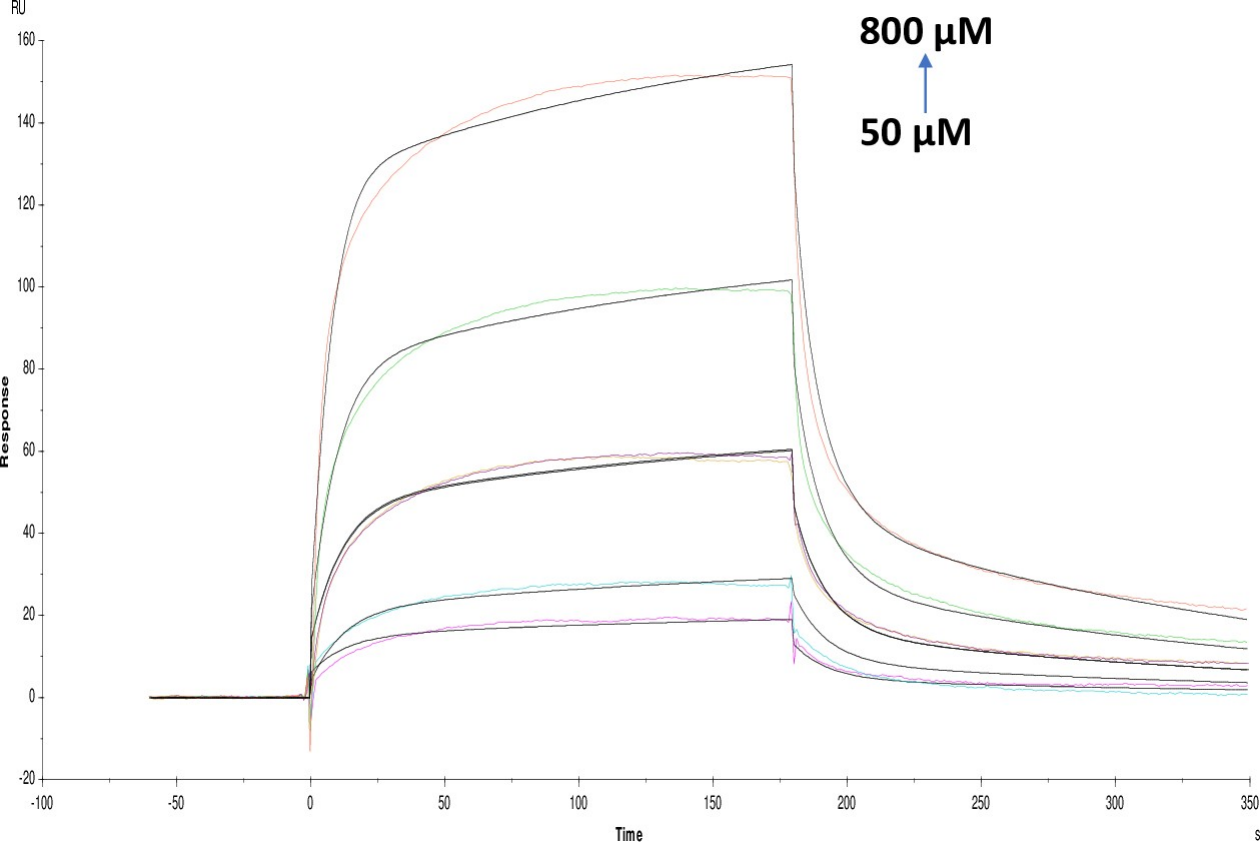
Binding sensorgram for Crocin interaction with immobilized AChE. Increasing concentrations of Crocin from 50 μM-800 μM were injected over the enzyme surface at 25°C. The flow rate is maintained at 45 μl min^-1^. Contact time and dissociation time was kept at 120 s and 200 s. Regeneration was carried out using 10 mM glycine HCl with pH 3 for 30 s and at 30 μl min^-1^. The data analysis was done using Biacore X100 evaluation software ver 2.0.2 and data was fit to two state. The resulting equilibrium dissociation constants KD, kinetic association kon and dissociation koff rates are given in Table 2.

**Fig 13:**
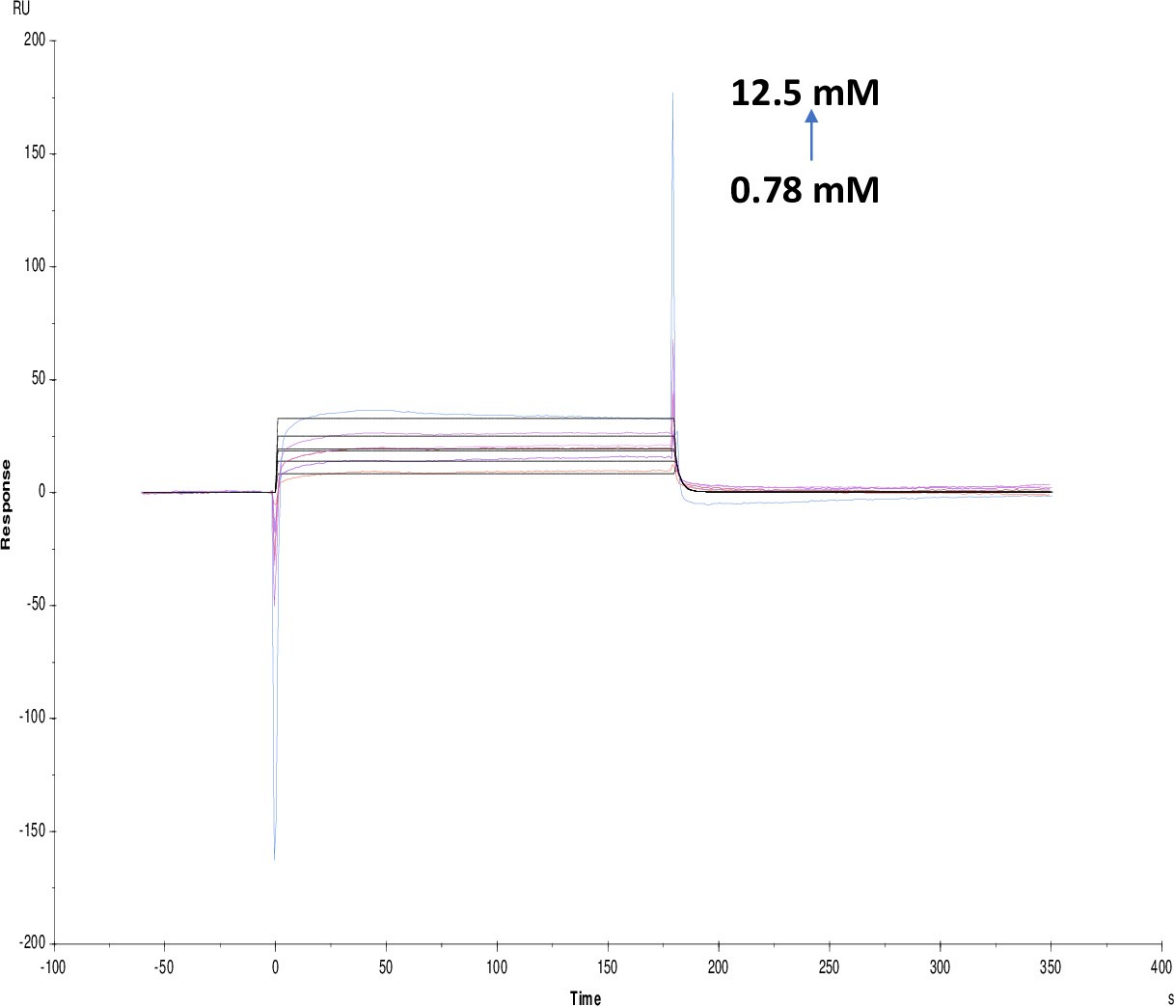
Binding sensorgram for ATCI interaction with immobilized AChE. Increasing concentrations of ATCI from 0.78 mM-12.5 mM were injected over the enzyme surface at 25°C. The flow rate is maintained at 45 μl min^-1^. Contact time and dissociation time was kept at 120 s and 200 s. Regeneration was carried out using 10 mM glycine HCl with pH 3 for 30 s and at 30 μl min^-1^. The data analysis was done using Biacore X100 evaluation software ver 2.0.2 and data was fit to two state. The resulting equilibrium dissociation constants KD, kinetic association kon and dissociation koff rates are given in Table 2.

**Fig 14:**
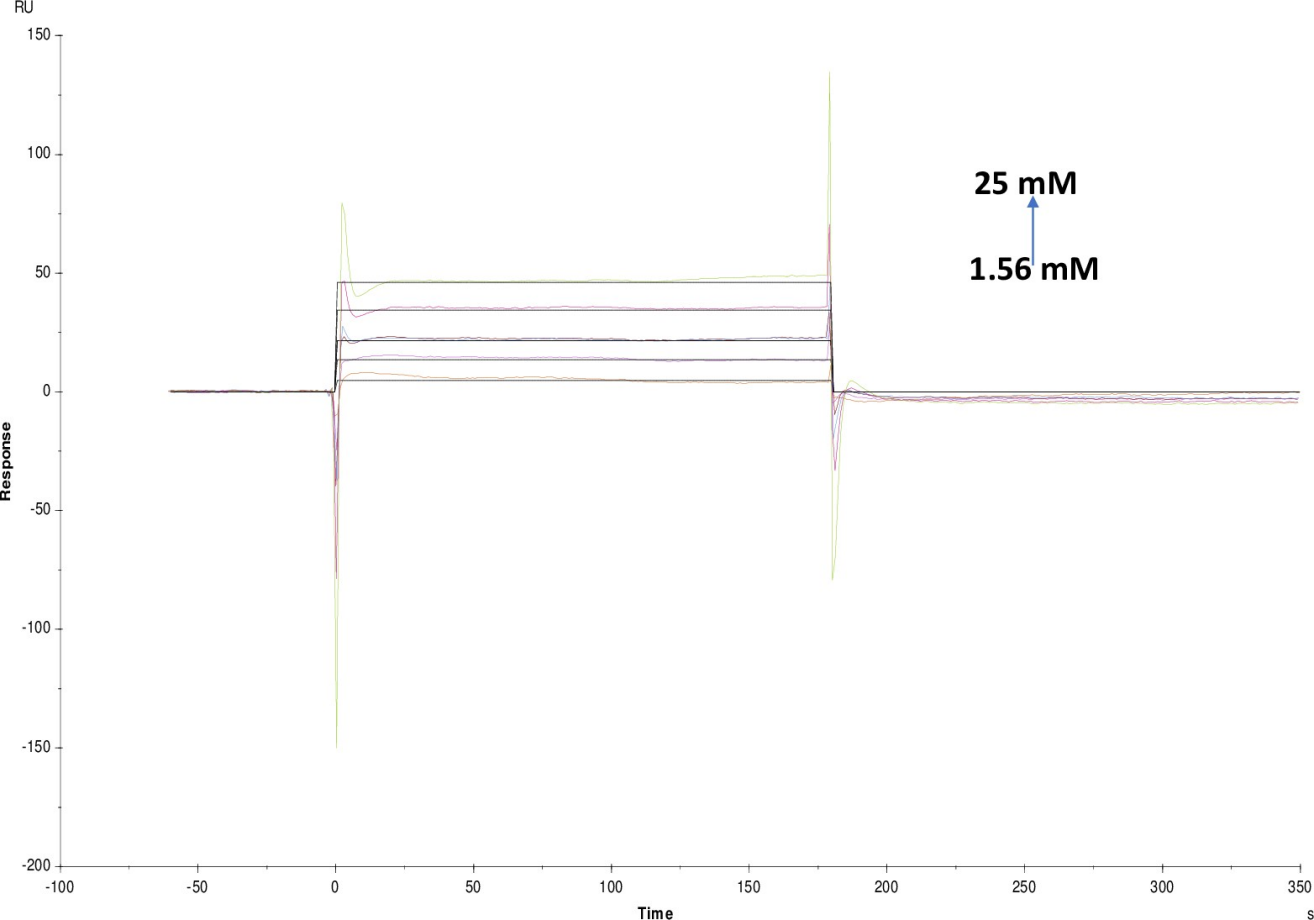
Binding sensorgram for carbachol interaction with immobilized AChE. Increasing concentrations of carbachol from 1.56 mM-25 mM were injected over the enzyme surface at 25°C. The flow rate is maintained at 45 μl min^-1^. Contact time and dissociation time was kept at 120 s and 200 s. Regeneration was carried out using 10 mM glycine HCl with pH 3 for 30 s and at 30 μl min^-1^. The data analysis was done using Biacore X100 evaluation software ver 2.0.2 and data was fit to two state. The resulting equilibrium dissociation constants KD, kinetic association kon and dissociation koff rates are given in Table 2.

Crocin, derived from the *Crocus sativus* stigmas is used as a tonic for the brain, nerve sedative and antidepressant. The SPR study of crocin was carried out which showed KD value of (4.702×10^-4^M) (Fig 12). Moreover, the affinity and kinetics of ATCI and carbachol were also studied where KD values were found to be (2.360×10^-7^M) and (2.235×10^-6^M) respectively (Figs 13 and 14). Where ATCI is a well-known substrate of AChE and injected repeatedly between different samples to check the stability of the immobilized AChE enzyme. Carbachol act as a substrate analogue [33] for AChE having the capacity to inhibit the enzyme, with a IC_50_ as reported in (Table 1). The tannic acid with an IC_50_ value of tannic acid 0.027±0.002 mM shows higher affinity as compared to small molecules as well as with standard inhibitor galanthamine hydrobromide. Also, affinity and kinetics of screened alkaloid and non-alkaloid molecules was found to be similar (Table 2).

**Table 2:** Interaction kinetics of AChE with different inhibitors fit to a two-state model using Biacore Evaluation Software version 2.0.

Molecules	K_a1_(1/Ms)	K_d1_(1/s)	K_a2_(1/s)	K_d2_(1/s)	KD(M) Kinetics	KD(M) Affinity
Galanthamine hydrobromide	347.8±13	0.5512±0.012	0.001879±4.2×10^−5^	0.003578×10^−4^	1.039×10^−3^	2.187×10^−3^
Galamer4	265.8±14	0.5171±0.013	8.446×10^5^±2.1×10^−5^	7.287×10^4^±0.0013	1.753×10^−3^	1.331×10^−3^
Caffiene	2.558×10^4^±2.4×10^3^	0.4490±0.012	2.705×10^6^±1.9×10^−5^	2.732×10^4^±1.7×10^−5^	1.738×10^−5^	7.701×10^−3^
Lycorine hydrochloride	131.5±6.3	0.3564±0.011	1.568×10^4^±2.9×10^−5^	0.02658±0.0061	5.533×10^−4^	1.715×10^−3^
Acetylthiocholineiodide (ATCI)	7.659×10^4^±1.2×10^4^	0.4167±0.13	1.244×10^4^±9.6×10^−5^	8.676×10^5^±0.0031	2.235×10^−6^	2.512×10^−3^
Carbachol	1.010×10^5^±9.7×10^3^	0.05155±0.0041	0.001509±2.6×10^−4^	0.001298±1.1×10^−4^	2.360×10^−7^	1.154×10^−3^
Quercetin	117.6±4.3	0.05923±0.0028	0.006822±3.4×10^−4^	0.004964±2.1×10^−4^	2.122×10^−4^	3.93×10^−4^
Catechin hydrate	76.33±3.4	0.06336±0.0042	0.01580±7.1×10^−4^	0.004173±1.1×10^−4^	1.734×10^−4^	7.38×10^−4^
Rutin hydrate	11.33±0.25	0.04254±0.0013	0.004468±1.6×10^−4^	0.002058±1.6×10^−4^	1.184×10^−3^	1.107×10^−3^
Pyrogallol	200.1±3.3	0.05082±0.0015	0.01979±3.7×10^−4^	0.002793±3.2×10^−5^	3.141×10^−5^	1.19×10^−4^
Tannic acid	1400±47	0.03423±0.017	0.1039±2.2×10^−4^	9.572×10^6^±3.4×10^−5^	2.250×10^−8^	1.140×10^−5^
Crocin/Saffran	6.017×10^5^±6.7×10^3^	472±5.2	0.003559±9.4×10^−5^	0.005325±1.5×10^−4^	4.702×10^−4^	4.75×10^−4^

Standard error values were calculated from three measurements for each analyte by the software itself

The data shown in Table 2 has a kinetic and binding affinity (KD) data. The kinetic data is represented by a binding phase denoted as ka1 and kd1 and a dissociation phase shown by ka2 and kd2. Two state binding models were used to evaluate the kinetic data. Two state models are used for cases where proteins undergo significant structural changes upon ligand binding. We have compared AChE secondary and tertiary structural changes in the presence of an analytes. SPR allows us to characterize small molecules based on the resident times or halflives so that the molecules serum half-life, the dosage can be calculated. This SPR sensor can detect various inhibitors with more accuracy and sensitivity in a single run. Also, these interactions are label-free and less error prone compared with other established spectroscopic methods [24]. The potency, kinetic and affinity binding parameters match for all the compounds studied.

**Fig 15:**
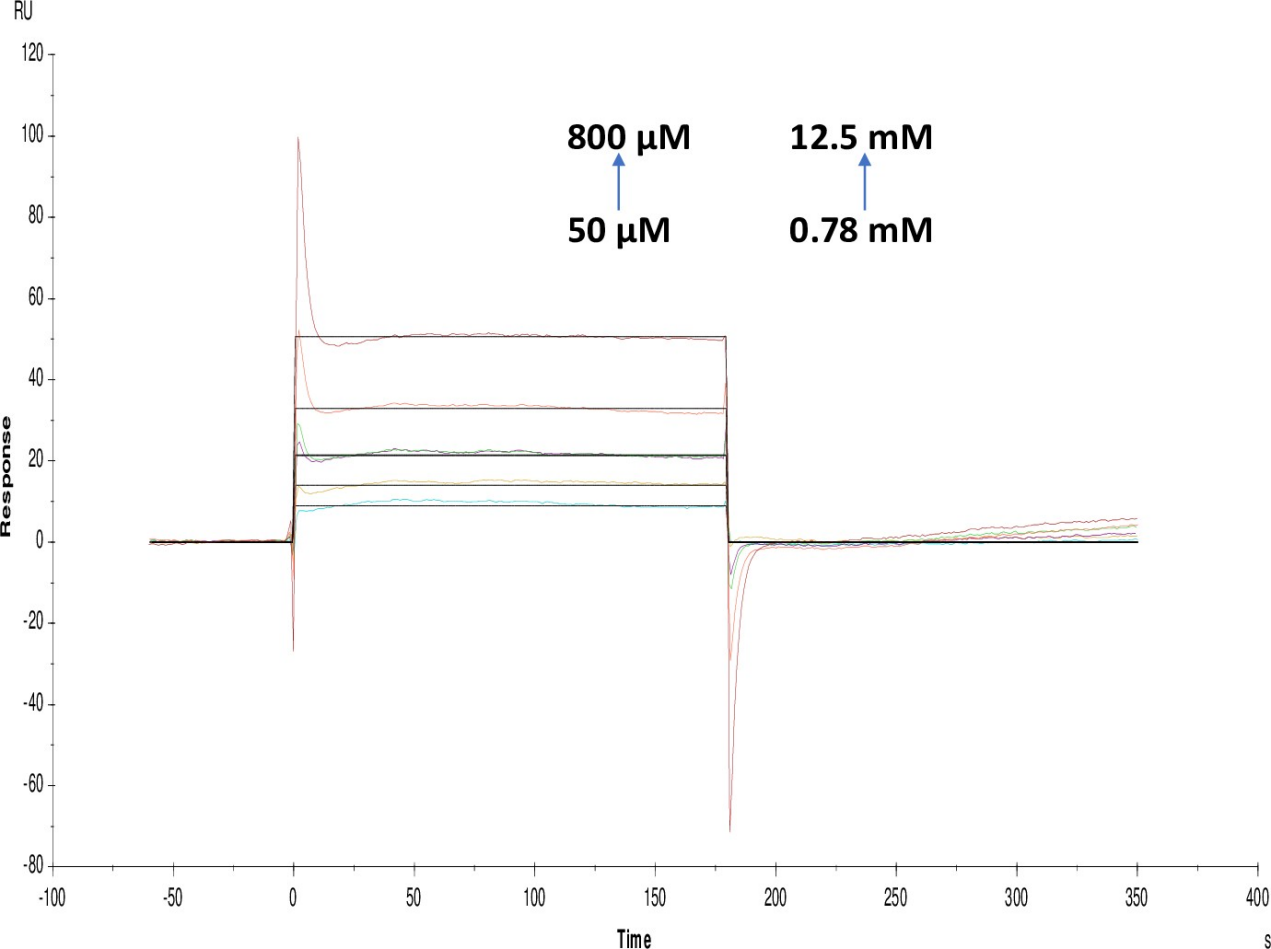
Binding sensorgram for galanthamine hydrobromide and ATCI interaction with immobilized AChE. Increasing concentrations of galanthamine hydrobromide from 50 μM-800 μM and ATCI from 0.78 mM-12.5 mM were injected over the enzyme surface at 25°C. The flow rate is maintained at 45 μl min^-1^. Contact time and dissociation time was kept at 120 s and 200 s. Regeneration was carried out using 10 mM glycine HCl with pH 3 for 30 s and at 30 μl min^-1^. The data analysis was done using Biacore X100 evaluation software ver 2.0.2 and data was fit to heterogeneous analyte fit. The resulting equilibrium dissociation constants are given in Table 3.

**Fig 16:**
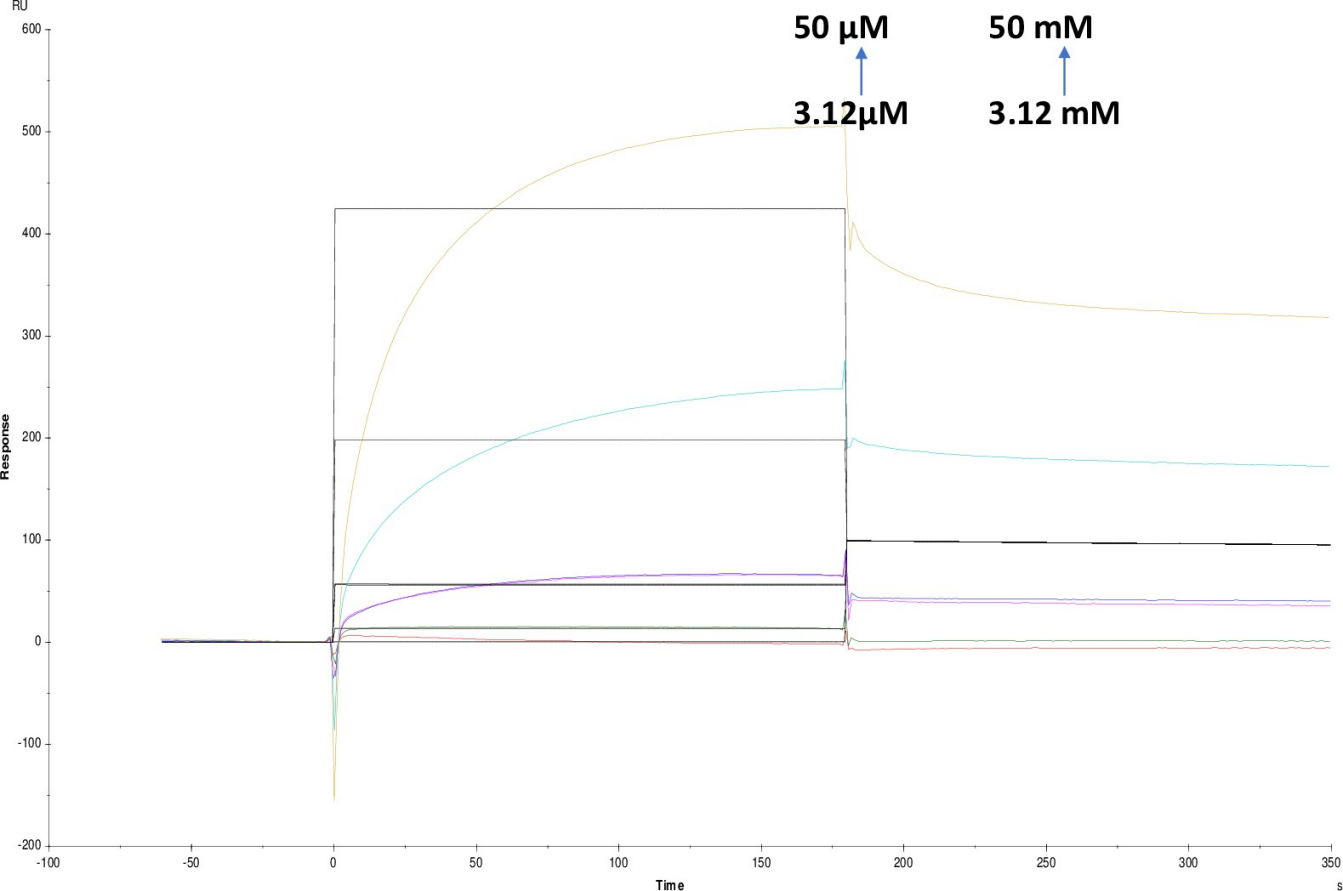
Binding sensorgram for tannic acid and ATCI interaction with immobilized AChE. Increasing concentrations of tannic acid from 3.12 μM-50 μM and ATCI from 3.12 mM-50 mM were injected over the enzyme surface at 25°C. The flow rate is maintained at 45 μl min^-1^. Contact time and dissociation time was kept at 120 s and 200 s. Regeneration was carried out using 10 mM glycine HCl with pH 3 for 30 s and at 30 μl min^-1^. The data analysis was done using Biacore X100 evaluation software ver 2.0.2 and data was fit to heterogeneous analyte fit. The resulting equilibrium dissociation constants are given in Table 3.

**Fig 17:**
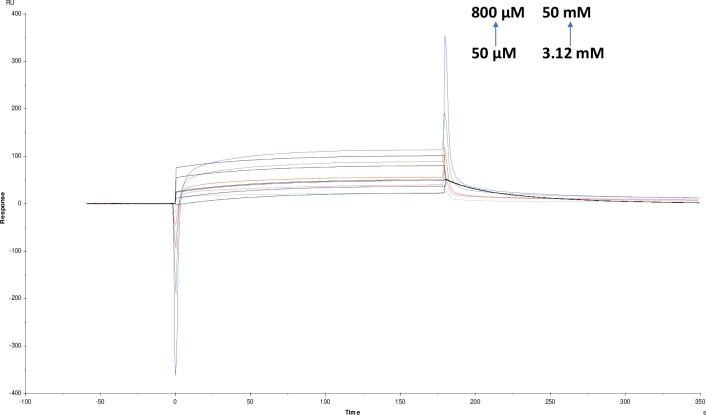
Binding sensorgram for crocin and ATCI interaction with immobilized AChE. Increasing concentrations of crocin from 50 μM-800 μM and ATCI from 3.12 mM-50 mM were injected over the enzyme surface at 25°C. The flow rate is maintained at 45 μl min^-1^. Contact time and dissociation time was kept at 120 s and 200 s. Regeneration was carried out using 10 mM glycine HCl with pH 3 for 30 s and at 30 μl min^-1^. The data analysis was done using Biacore X100 evaluation software ver 2.0.2 and data was fit to heterogeneous analyte fit. The resulting equilibrium dissociation constants are given in Table 3.

**Fig 18:**
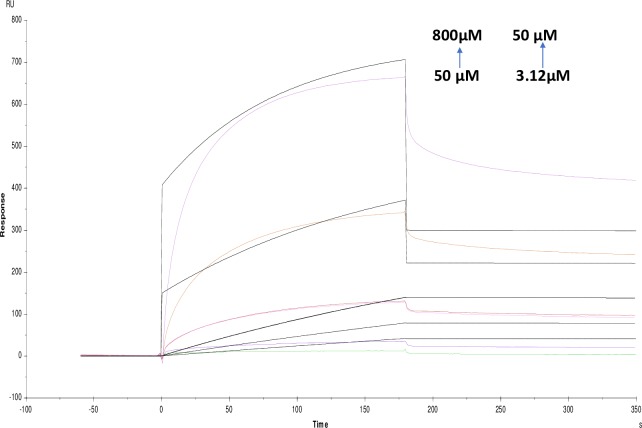
Binding sensorgram for tannic acid and galanthamine hydrobromide interaction with immobilized AChE. Increasing concentrations of tannic acid from 3.12 μM-50 μM and galanthamine hydrobromide from 50 μM-800 μM were injected over the enzyme surface at 25°C. The flow rate is maintained at 45 μl min^-1^. Contact time and dissociation time was kept at 120 s and 200 s. Regeneration was carried out using 10 mM glycine HCl with pH 3 for 30 s and at 30 μl min^-1^. The data analysis was done using Biacore X100 evaluation software ver 2.0.2 and data was fit to heterogeneous analyte fit. The resulting equilibrium dissociation constants are given in Table 3.

**Fig 19:**
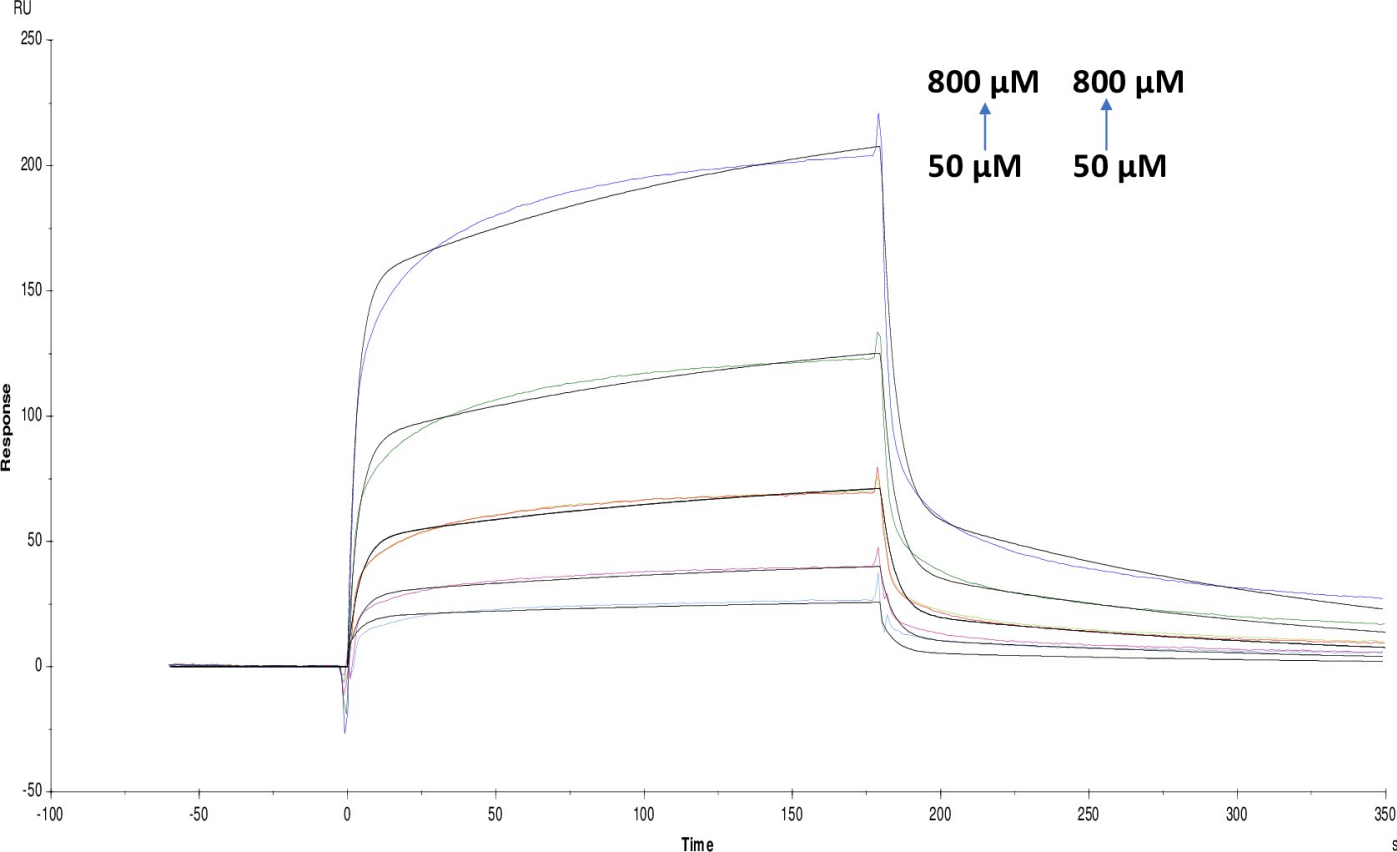
Binding sensorgram for galanthamine hydrobromide and crocin interaction with immobilized AChE. Increasing concentrations of galanthamine hydrobromide from 50 μM—800 μM and crocin from 50 μM-800 μM were injected over the enzyme surface at 25°C. The flow rate is maintained at 45 μl min^-1^. Contact time and dissociation time was kept at 120 s and 200 s. Regeneration was carried out using 10 mM glycine HCl with pH 3 for 30 s and at 30 μl min^-1^. The data analysis was done using Biacore X100 evaluation software ver 2.0.2 and data was fit to heterogeneous analyte fit. The resulting equilibrium dissociation constants are given in Table 3.

Likewise, competitive experiments between different pairs was run by mixing 1:1 ratio of molecules and was fit to heterogeneous analyte model were studied. These experiments are helpful for determining the kinetics of a small molecule analyte in competition with other. Response contributions from both analytes are taken into account by considering molecular weight and concentrations of analytes [26]. For competition binding experiments, pairing was done based on substrate-inhibitor and inhibitor-inhibitor interactions. Among screened molecules, tannic acid, crocin, galanthamine hydrobromide and ATCI were further selected for the competition studies. These molecules were chosen because galanthamine hydrobromide is a potent inhibitor, and ATCI is a well-known substrate and tannic acid as it showed highest affinity and kineticsin our studies. Finally, naturally occurring crocin was also tested due to reports in the literature as a brain tonic for the Alzheimer's [34]. In the competition studies, galanthamine hydrobromide and ATCI showed KD (equillibrium dissociation constant) values as (9.651×10^-9^M) and (9.892×10^-9^M) respectively. For tannic acid and ATCI, KD was (7.507×10^-8^M) and (1.597×10^-10^M) and finally for crocin and ATCI were it showed KD as (4.624×10^-7^M) and (5.513×10^-6^M) (Figs 15–17) (Table 3). Taken together, there is a considerable change in KD values compared with individual one. In case of inhibitor-inhibitor competition studies with galanthamine hydrobromide and crocin, KD value obtained was (1.740×10^-3^M) and (6.022×10^-3^M); for galanthamine hydrobromide and tannic acid were (2.910×10^-3^M) and (1.162×10^-7^M) respectively (Figs 18 and 19). However, in the case of two inhibitors, KD was higher as compared with an individual molecules resulting in a lower affinity for the ligand. Additionally, the affinity for tannic acid and crocin were lower as compared to individual experiments. This might be due to two inhibitors competing with each other for binding towards enzyme which is also supported by modeling data.

**Table 3:** Interaction kinetics of AChE with different heterogeneous analytes fit a heterogeneous analyte fit using Biacore Evaluation Software version 2.0.

Molecules	Molecule 1			Molecule 2		
	ka1(1/Ms) [millisecond]	kd1(1/s)	KD(M)	ka2(1/Ms)	kd2(1/s)	KD(M)
**Galanthamine hydrobromide (molecule 1) and ATCI (molecule 2)**	1.174×10^5^±4.8×10^4^	0.001133±5×10^−4^	9.651×10^−9^	9.291×10^4^±4.3×10^4^	9.191×10^4^±0.0013	9.892×10^−9^
**Tannic acid(molecule 1) and ATCI (molecule 2)**	1.687×10^−5^±3.7×10^6^	0.01266±0.028	7.507×10^−8^	1.529×10^6^±3.4×10^6^	2.441×10^4^±2.8×10^−4^	1.597×10^−10^
**crocin (molecule 1) and ATCI (molecule 2)**	3281±5.8×10^2^	0.01684±8.5×10^−4^	4.624×10^−7^	1.395×10^5^±1.9×10^4^	0.06452±0.0034	5.513×10^−6^
**Tannic acid (molecule 1) and Galanthamine hydrobromide (molecule 2)**	231.1±3.3	2.685×10^−5^±2.9×10^−8^	1.162×10^−7^	0.06842 ±0.093	1.991×10^4^±1.2×10^−7^	2.910×10^−3^
**Galanthamine hydrobromide (molecule 1) and crocin (molecule 2)**	127.3±5.0	0.221±0.0044	1.740×10^−3^	1.010±0.11	0.006083±1.1×10^−4^	6.022×10^−3^

Standard error values were calculated from three measurements for each analyte by the software itself

Biacore based biosensor technology is widely accepted technology for molecular interactions. An in depth kinetic characterization of small molecules can be possible based on this technology. Surface competition experiments can give a better affinity data for compound target interaction [35].

### Circular dichroism studies

**Fig 20:**
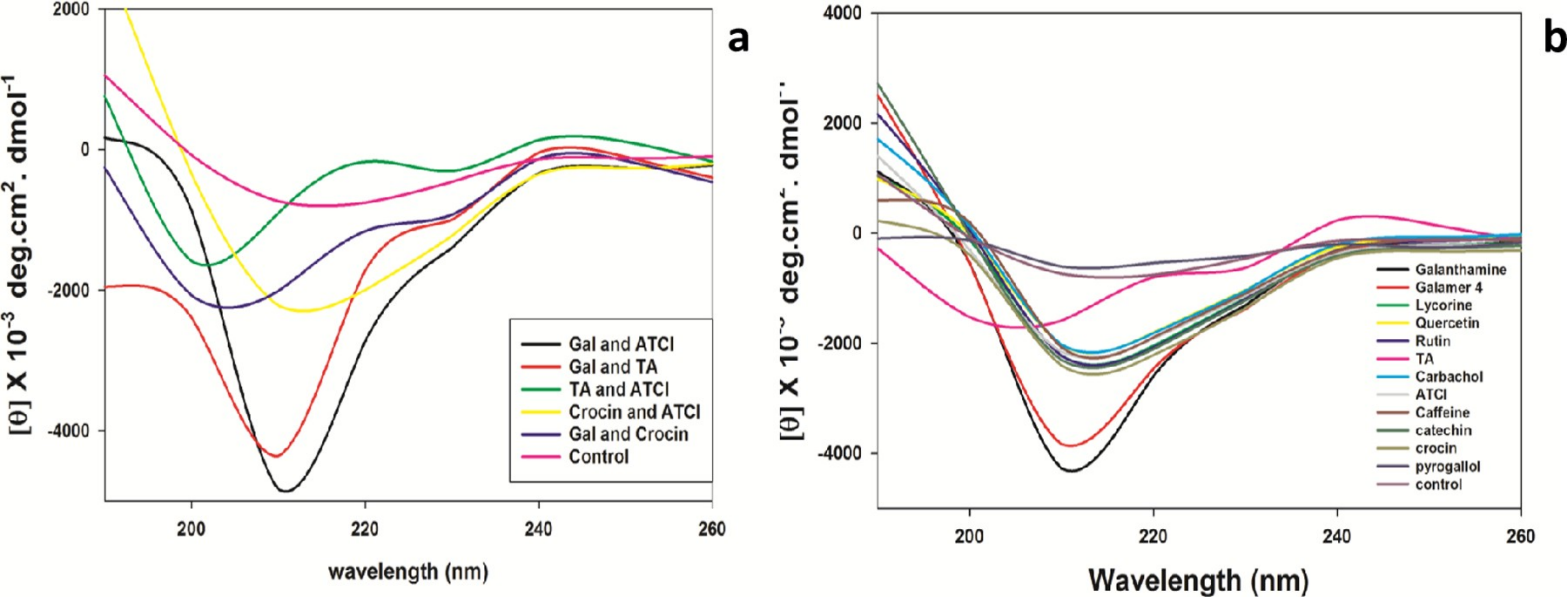
**a)** Circular dichroism (CD) spectropolarimetry. Far-UV CD spectra of AChE in the presence of heterogeneous analytes as, (galanthamine hydrobromide and ATCI), (tannic acid and ATCI), (crocin and ATCI) and inhibitor-inhibitor (galanthamine hydrobromide and tannic acid) (galanthamine hydrobromide and crocin). The CD data were expressed as molar ellipticity (deg cm^2^ dmol^-1^). **b)** Circular dichroism (CD) spectropolarimetry. Far-UV CD spectra of AChE in the presence of galanthamine hydrobromide (Gal), galamer4, caffeine, lycorine hydrochloride, crocin, rutin hydrate, pyrogallol, quercetin, catechin hydrate, tannic acid (TA), ATCI and carbachol. The CD data were expressed as molar ellipticity [degree centimeter square degree per molar (deg cm^2^ dmol^-1^)].

CD studies showed significant changes in the secondary structure of AChE in presence of inhibitors. The CD spectra exhibited two minima at 208 nm and 222 nm which is the characteristic feature of the class of proteins. It was noted that amongst alkaloids studied with AChE, there is a decrease in α-helixe compared to control enzyme while an increase of α-helix in case of lycorine hydrochloride. In the case of caffeine, there was a considerable change in β-sheets. Amongst phenolics, only quercetin showed a reduction in α-helix with an increase in β-sheets. For the remaining molecules, there was a significant increase in α-helix and a considerable reduction in β-sheets. Finally, AChE in the presence of substrates like ATCI and substrate analogue carbachol was showing an increase in α-helix and a decline in β-sheets. In combination studies, inhibitor-substrate and inhibitor-inhibitor there were remarkable changes in the secondary structure of the protein. The % changes with and without inhibitors are mentioned in (Tables 4 and 5) (Fig 20). This shows AChE undergoes changes in presence of analytes studies. Also reason for the presence of gorge in the molecule and penetration of compounds to the active catalytic site [36].

**Table 4:** Effect of different small molecules on secondary structure composition of AChE in presence and absence of small molecules.

Molecule	α helix%	β sheets%	Turns%	Random%
AChE	3.6±0.012	52.3±0.6	0	44.2±0.46
Galanthamine hydrobromide	0	46±0.50	7.6±0.06	46.4±0.52
Galamer 4	0.6±0.001	46.8±0.53	6.6±0.04	46±0.49
Caffiene	1.2±0.009	54.5±0.8	1.6±0.010	42.7±0.39
Lycorine hydrochloride	7.2±0.045	51.2±0.72	0	41.6±0.34
Carbachol	4.0±0.02	51.0±0.69	2.6±0.016	42.4±0.38
ATCI	5.9±0.035	51.3±0.70	0	42.8±0.39
Quercetin	3.1±0.010	53.2±0.75	1.0±0.006	42.8±0.39
Catechin hydrate	10±0.06	49±0.67	0	41±0.29
Rutin hydrate	8.8±0.056	50.5±0.72	0	40.7±0.27
Pyrogallol	0	50.8±0.73	0	49.2±0.68
Tannic acid	5.1±0.039	30.6±0.19	14.8±0.08	49.5±0.70
Crocin	5.7±0.042	50.5±0.74	0	43.8±0.41

Standard error values were calculated from three measurements for each analyte by the software itself

**Table 5:** Effect of two small molecules on secondary structure composition of AChE in presence and absence of small molecules.

Molecule	α helix %	β sheets%	Turns%	Random%
Galanthamine hydrobromide and ATCI	0	45.2±0.5	7.5±0.06	47.3±0.59
Tannic acid and ATCI	16.9±0.2	0	27.3±0.32	55.7±0.72
Crocin and ATCI	4.3±0.03	51.3±0.65	0	44.4±0.43
Tannic acid and Galanthamine hydrobromide	0	39.1±0.31	10.3±0.09	50.6±0.51
Galanthamine hydrobromide and crocin	6.3±0.06	32.9±0.31	8.2±0.08	52.6±0.69

Standard error values were calculated from three measurements for each analyte by the software itself

### Fluorescence spectral studies

**Fig 21:**
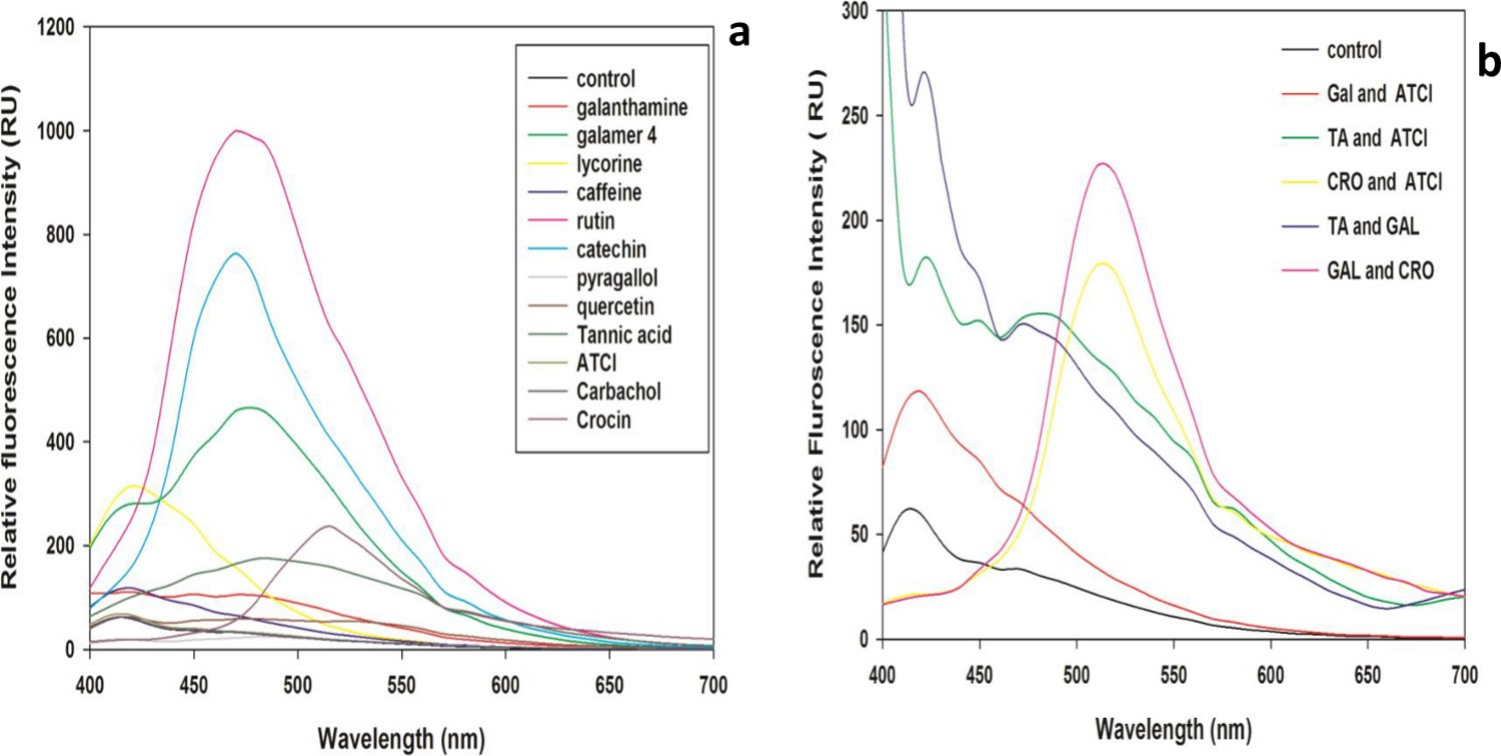
**a)** Intrinsic fluorescence spectra of the AChE incubated with molecules. The protein solutions of 0.5 mg ml^−1^ were prepared in 10mM PBS buffer at pH 7.7 and incubated with galanthamine hydrobromide, galamer4, caffeine and lycorine hydrochloride, crocin, rutin hydrate, pyrogallol, quercetin, catechin hydrate and tannic acid, ATCI and carbachol at different concentrations each for 10 min, at 37°C and used to obtain the spectra. Scans were performed at 37°C using a 1 cm path length quartz cuvette with 1 s differential integration time at a scan rate of 600 nm min^-1^. The excitation wavelength was 365 nm and emission wavelength was in the range of 400–700 nm. **b)** Intrinsic fluorescence spectra of the AChE incubated with molecules. The protein solutions of 0.5 mg ml^−1^ were prepared in 10 mM PBS buffer at pH 7.7 and incubated with (galanthamine hydrobromide [Gal] and ATCI), (Tannic acid [TA] and ATCI), (crocin [Cro] and ATCI) and inhibitor-inhibitor (galanthamine hydrobromide and tannic acid) (galanthamine hydrobromide and crocin) at different concentrations each for 10 min, at 37°C and used to obtain the spectra. Scans were performed at 37° C using a 1 cm path length quartz cuvette with 1 s differential integration time at a scan rate of 600 nm min^-1^. The excitation wavelength was 365 nm and the emission wavelength was in the range of 400–700 nm.

The fluorescence studies revealed that few molecules were showing higher intensity compared to control and few were showing less intensity including a shift in the maximum intensity as compared with the control enzyme. The emission spectrum of control protein showed emission maxima at 415 nm. There was a change in the intensity for different molecules. In this case, galanthamine hydrobromide, caffeine, lycorine hydrochloride was showing an increase in the intensity at 415 nm. While in the case of galamer4, rutin hydrate, catechin hydrate, quercetin, tannic acid and crocin displaying a redshift but pyrogallol was exhibiting quenching as compared to control enzyme. Substrates like carbachol and ATCI were indicating no change when compared with control enzyme. In the case of a combination of molecules, galanthamine hydrobromide and ATCI, tannic acid and ATCI and finally, galanthamine hydrobromide and tannic acid were resulting in significant increase in the intensity, whereas galanthamine hydrobromide and crocin, crocin and ATCI there was a red shift in emission maxima as compared with control enzyme. There were prominent changes in the fluorescence spectra of AChE in presence of compounds perhaps due to reorganization of tertiary structure or conformational changes in the enzyme due to compound binding (Fig 21).

### Molecular modeling studies

**Fig 22:**
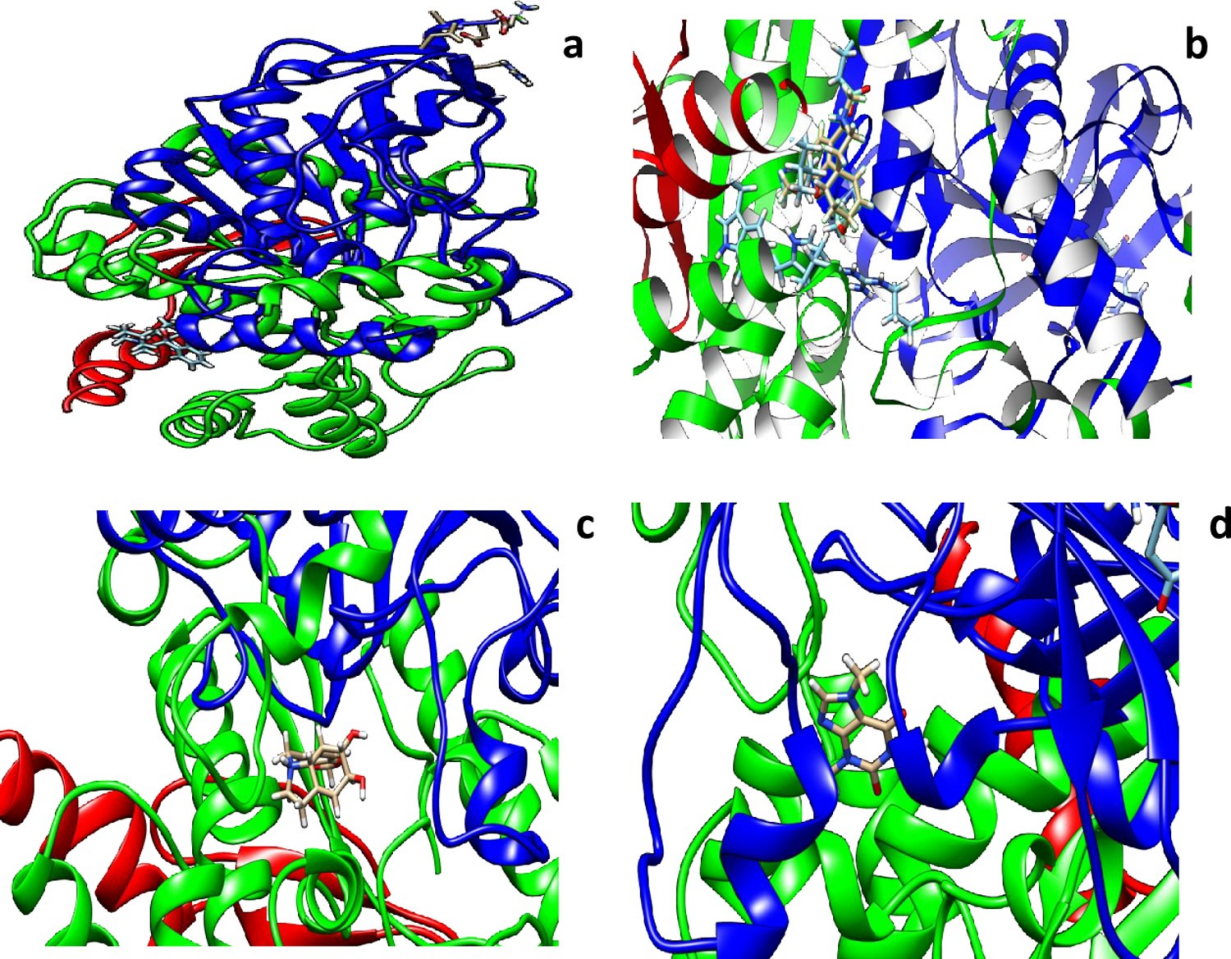
In silico docking of crystal structure of human recombinant AChE (4EY7) with different molecules **a)** galanthamine hydrobromide **b)** galamer4 **c)** lycorine and **d)** caffeine were used in these docking studies. All the molecules were taken from Zinc database. UCSF Chimera 1.12 software was used for visualization of the results and creating images.

**Fig 23:**
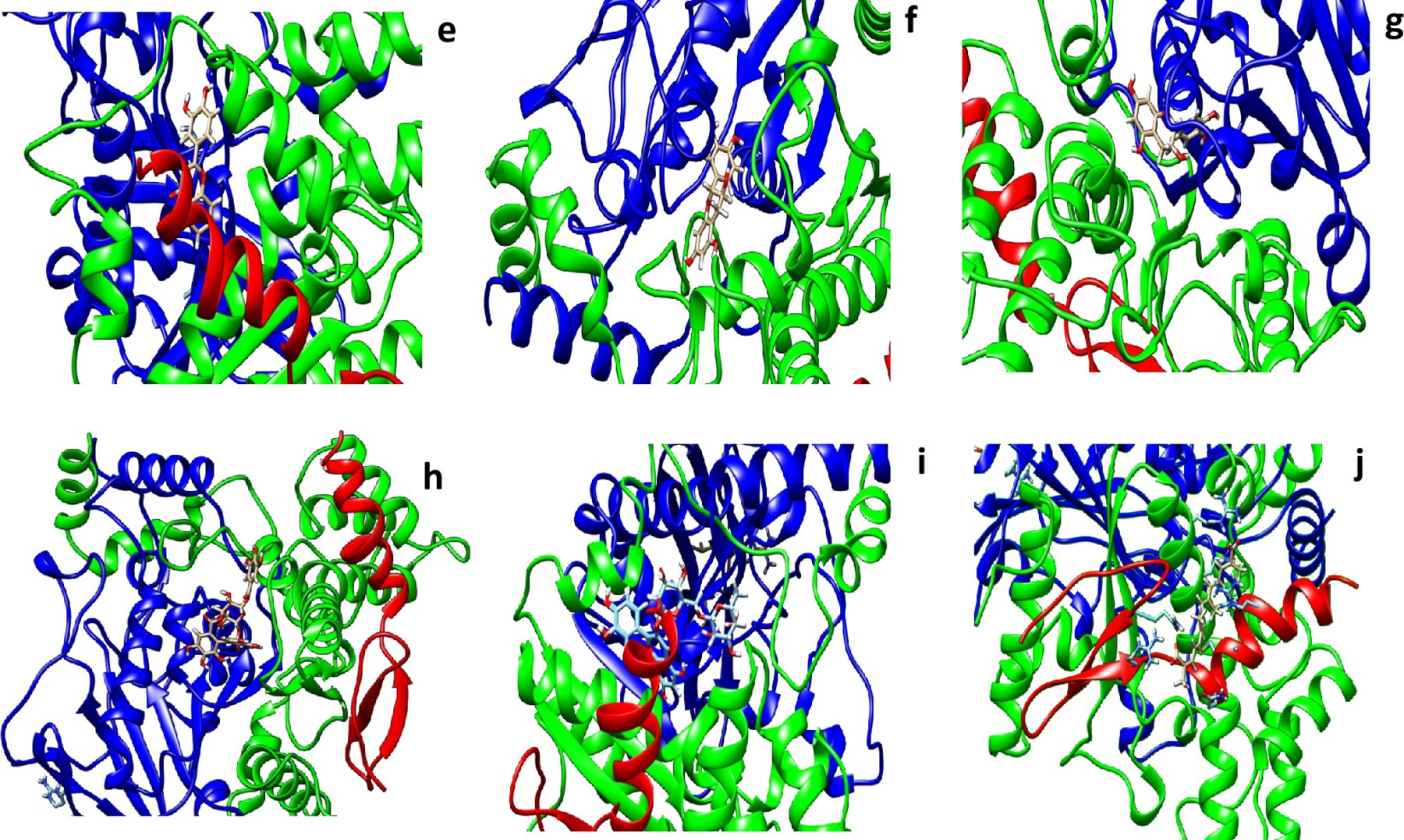
In silico docking of crystal structure of human recombinant AChE (4EY7) with different molecules **e**) quercetin **f)** catechin hydrate **g)** pyrogallol **h)** tannic acid **i)** rutin hydrate and **j)** crocin were used in these docking studies. All the molecules were taken from Zinc database. UCSF Chimera 1.12 software was used for visualization of the results and creating images.

**Fig 24:**
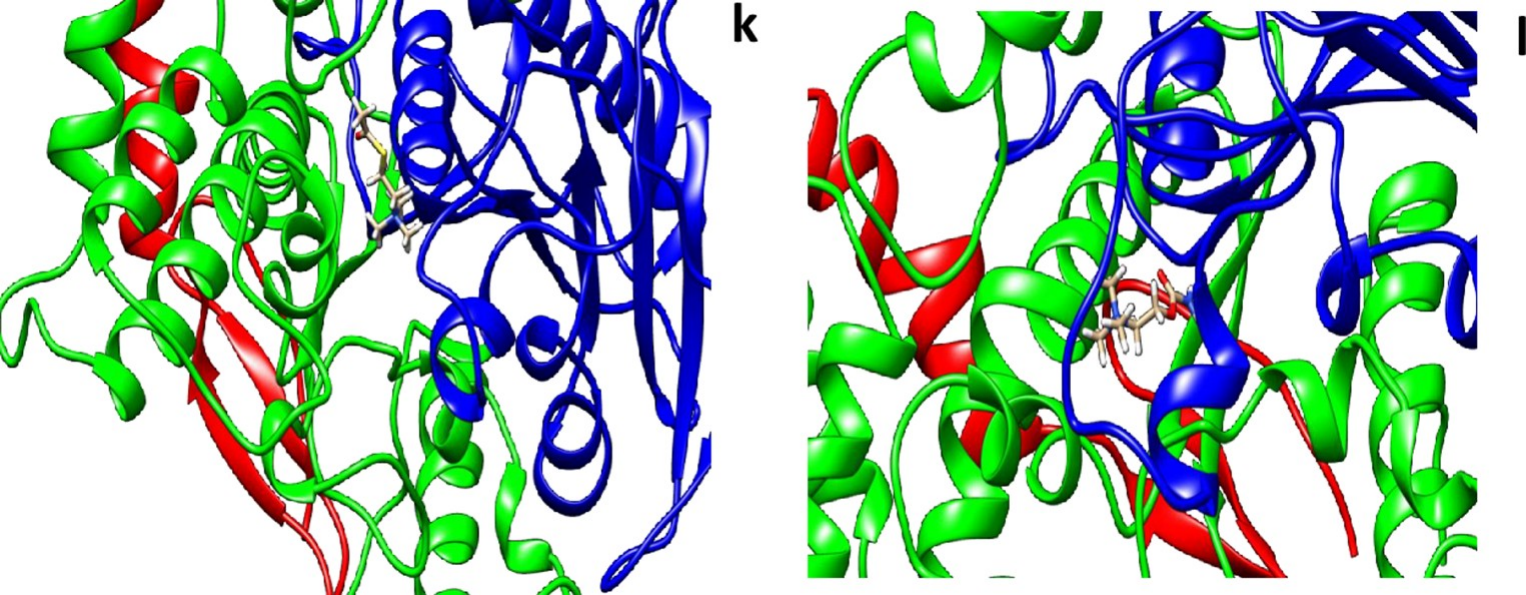
In silico docking of crystal structure of human recombinant AChE (4EY7) with different molecules **k)** ATCI and **l)** carbachol were used in these docking studies. All the molecules were taken from Zinc database. UCSF Chimera 1.12 software was used for visualization of the results and creating images.

The crystal structure of human AChE at a resolution of 2.35 Å was downloaded from the PDB (PDB entry: 4EY7) and was used as the initial 3D model. To obtain the docking–binding models for AChE in complexes with G1 and G2, the molecular modeling program swiss dock was used to perform the docking process. Interactions of various small molecules were studied with AChE using Swiss dock server. The lowest binding energy as tabulated in (Table 6) along with the residues involved in the interaction. All the molecules exhibited low binding energy of interaction in forming stable bonds. We used biperidine as a standard molecule for docking. Biperidine was published in the literature and shown to have -7.9 kCalmol^-1^ [37]. In our present studies, we also got similar binding energy. The amino acids tabulated against each molecule have interaction energies below -0.4kCalmol^-1^. Generally, if the interaction energy between a residue and a ligand is lower than -0.8kCalmol^-1^, the residue is regarded as an essential residue in the molecular recognition of that ligand (Figs 22, 23 and 24).

**Table 6:** Docking studies of different small molecules interacting with human recombinant AChE (4EY7).

Sl.No.	Molecules	Binding free energy by modelling ΔG (kcal/mol)	Amino acids involved in the interactions
1	Galanthamine hydrobromide	-7.7018876	HSD 405;GLU 313;ARG 296
2	Galamer 4	NA	NA
3	Caffiene	-6.8859744	TYR86;SER125;HSD447;GLY121;GLY120;TYR337;GLU202;GLY448;GLY126
4	Lycorine hydrochloride	-7.8588786	PHE295;TYR72;VAL294;TRP286;SER293;TYR341;PHE297
5	Acetylthiocholineiodide (ATCI)	-7.7025747	PHE295;TYR341;PHE338;TYR124TYR337;PHE297
6	Carbachol	-7.5991077	Glu202; TYR337; TRP 86; TYR124; TRP86;PHE338; GLY 448; HSD 447;
7	Quercetin	-7.460357	PRO368;ASN533;HSD405;ARG296;TRP532;PRO235;PRO235;VAL370;LEU540;PRO410;ASN533
8	Catechin hydrate	-7.6887374	GLU202;GLY121;HSD447;PHE338;SER203;TYR 124;TYR337;TYR 124;TYR341
9	Rutin hydrate	-9.214323	Pro 368;Arg 296;Pro 537; HSD 405; Glu313; TRP 532
10	Pyrogallol	-6.846631	GLU202;TYR341;TYR133;TYR 337;TYR 124;PHE 338;PHE 297;GLY 121;GLY 120;HSD 447;SER 203;TRP86
11	Tannic acid	-7.733458	ARG219;ASP320;ALA318;GLY319HSD322
12	Crocin	-10.531265	GLU202;PHE 338;PHE297;TYR 337;TYR 341;TYR133;TYR124;TRP86;GLY121;HSD447;SER203
13	^*^Biperidin (Standard for docking)	-7.92	NA

* Standard molecule for docking studies

## Discussion

New research is emerging on the role of cholinergic drugs in the treatment of many other diseases like dementia [38], multiple sclerosis [39], tobacco and alcohol usage disorder [40,41] nausea, opioid use disorder or opioid overdosage and cocaine addiction [42]. Anticholinergic drugs or inhibitors differ in their pharmacological profile and warrants more research into their dosing, PK (Pharmacokinetic), PD (Pharmacodynamic) and considerable side effects [43]. Mainly AChEI is also used clinically for the treatment of various diseases like glaucoma, myasthenia gravis and Lewy body dementia. For the effective treatment, potent inhibitors must bind to the active site of the enzyme reversibly, as irreversible binding may cause severe consequences, eventually loss of receptor activity and significant toxicological effects [5]. Hence, reversible AChE inhibitors have become the increasingly central role for the treatment of AD. Also, natural molecules and plant secondary metabolites are found to be superior in their inhibiting potential compared to synthetic molecules [44]. Nowadays, research is mainly focussed on natural molecules from plants as prominent sources of new or rather more effective AChEI. Hence, in present study, the screening of binding kinetics and competitive studies of various natural small molecules against AChE enzyme have been evaluated. Whereas, the previous studies were available only on the synthetic drug molecules inhibiting AChE. But comparatively very few studies have been reported on the characterization and dosing of inhibitors by using SPR.

Different plant families have been reported in traditional medicinal practices having the potential to inhibit AChE. Families like Acanthaceae, Amaranthaceae, Amaryllidaceae, Anacardiaceae, Apiaceae, Illiciaceae, Lamiaceae, Zingiberaceae, Solanaceae, Rutaceae and Polygonaceae are reported for having AChE inhibitory activities [45,46] due to its secondary metabolites contents. New data is emerging in addition to already existing reports on alkaloid namely galanthamine [47]. Galanthamine in combination with methadone can have an additive effect in patients treated with heroin overdose [42]. Earlier report suggested that coadministration of caffeine with donepezil had a better effect on AChE inhibition [48] also, another alkaloid lycorine and its derivatives isolated from the Amaryllidaceae family were found to be effective inhibitor however, they are not taken further to clinics [49]. In our studies, it was observed that among the compounds screened, galanthamine has a low IC_50_ and affinity compared to other alkaloids, in contrast caffeine has high affinity. Taken together potency and affinity can predict clinical efficacy.

Similarly, many non-alkaloid molecules from natural sources like polyphenols, flavonoids, tannins, anthocyanidins are potent AChEIs and perhaps play a vital role as an antioxidant and robust metal chelator which may contribute in reducing oxidative stress of the tissue affected in AD [17, 5]. Among the flavonoids, quercetin from *Polygonum sachalinensis* is having good antioxidant, anti-inflammatory ability and AChE inhibition activity [20]. Different flavonoids had the ability to exhibit the highest cholinesterase inhibition activities [17]. Among flavanols catechin isolated from *Eugenia dysenterica* is having IC_50_ of 42.39 μgml^-1^ [50] and epigallocatechin gallate having IC_50_ value of 16.83 μM. Also, *Ganoderma alucidum* extract has AChE inhibition due to its valuable phenolic antioxidants like quercetin, catechin, and rutin [51]. Catechin isolated from *Dasiphora fruticosa* shows AChE inhibition with IC_50_ 1.042mM [52]. Pyrogallol a prominent polyphenol found in various fruits and vegetables was able to observe as an effective AChEI showing the IC_50_ value of 10.22 μM [53]. Some studies on plant extract of *T*. *chebula* having active constituent tannic acid was exhibiting potential to inhibit AChE [54] and also 0.04 mM tannic acid reduced 37% AChE activity of the central nervous tissue of *L*.*acuminata* [55]. *Bouvardia ternifolia* revealed good AChE inhibition due to polyphenols existing in the extract and rutin hydrate was one of the polyphenol able to induce AChE inhibition [56]. Rutin from *Hypericum* species has shown AChE inhibition. [5].[34] Safranal, crocin, and dihydrocrocin were able to inhibit AChE where safranal is showing maximum inhibition with 21.09 ± 0.17μM concentration.

In the current studies among the non-alkaloids, tannic acid is having high potency and slow rate of dissociation as compared with other small molecules. Slow dissociation of a molecule from target or high residence time shows better efficacy in clinics [57]. Milkani et al [21] have studied the interaction of small molecules with AChE enzyme and enzyme integrity after immobilization on an SPR sensor chip was determined by using a photometric assay. They reasoned that the molecules bring in conformational changes upon binding to AChE. Also, immobilization of recombinant human AChE on SPR chip was studied with four known ligands and compared to human serum albumin thereby correlated to the ADME data [22]. Additionally, to determine the kinetic affinity precisely we have used the competitive binding mode of experiments. With the affinity information, if two molecules are known a competition experiment can be set up thereby the kinetic KD can be determined accurately. Such experiments decipher the drug-drug interactions and also drug additive effects.

CD was used for investigating changes in secondary structure of the protein in presence of small molecules. Previous CD studies on AChE showed changes in the structure of the enzyme in presence of edrophonium and propidium [58]. The transition from the α helix to β sheets was studied on AChE [59], where they observed that sodium selenate can affect the structure and dynamics of the active site gorge of AChE. In the presence of floribundiquinone B, isolated from the roots of *Berchemia*, CD transitions were observed from α helix to β turn, and random coil and β folds were significantly decreased. In addition to above mentioned studies in fluorescence study it was observed that, dose dependent quenching of intensity of AChE as a function of floribundiquinone B concentration was determined [60]. Also, the interaction of berberine with AChE was examined by fluorescence and CD studies to check the conformational and tertiary structure changes in the enzyme [61]. Above mentioned studies, support our CD and fluorescence data and expressed significant perturbations in the secondary and tertiary structure of the acetylcholinesterase. Studies on the crystal structures have shown a 20A° active site gorge with aromatic amino acids adjacent to it. Binding of molecules to the catalytic triad site requires them to enter into the gorge and perhaps the reason for spectral perturbations [62].

Molecular modeling or *in silico* docking prediction of the molecules binding to AChE displayed binding energies comparable in range of -6.8 to 7.8 kCal^-1^. For molecules like rutin and crocin are slightly higher in the range of -8.7 to -10.2. Also for the consistency with the published literature and having a positive control we used the biperidine binding to AChE and the binding energy data of -7.9 kCal^-1^ is also comparable and stands as a positive control. We presented the residues within 4.0 Å to the ligands and tabulated in the (Table 4). All the amino acids fall into two clusters with coordinates with X,Y,Z centers 1 [-11.241,-44.231,30.315] and 2 [-0.685, -39.128, 12.355]. We independently confirmed the clusters using Site Hound (http://scbx.mssm.edu/sitehound//sitehound-web/Input.html) web-based ligand binding sites prediction software. Most of the amino acids in these clusters match the chimera of predicted amino acids closer to ligands. Docking studies also support the spectral changes observed by CD and fluorescence experiments.

According to the current scenario, natural products like polyphenols exhibit minimum complications in comparison to other synthetic drug molecules and can be beneficial for impeding the progression of AD. In these current studies, it is proved that polyphenols are promising molecules to inhibit AChE in comparison with alkaloids. Taking together, AChE exhibits different affinities for various molecules, amongst those tannic acid resulted in highest affinity followed by carbachol, ATCI, pyrogallol, caffeine, crocin, quercetin, catechin hydrate, lycorine hydrochloride, rutin hydrate, galamer4 and galanthamine hydrobromide. Overall resilts concluded that, kinetic and affinity of all molecules were exhibiting similar KD and these data has been supported by various biophysical methods mentioned above. In the case of competitive analyte binding studies both the analytes were found to have higher or lower affinities in comparison with individual ones [63]. However, we did not use this data to understand the active site interaction information. On the basis of that one can compare low affinity ligands and also understand their active site binding. Thus in conclusion, SPR biosensor based studies and biophysical characterization of small molecules in presence of AChE can be further helpful for drug discovery against AD.

## Abbreviations

AD: Alzheimer’s Disease
Ach: Acetylcholine
AChE: Acetylcholinesterase
AChEI: Acetylcholinesterase inhibitor
ATCI: Acetylthiocholineiodide
DTNB: 5,5'-dithiobis-(2-nitrobenzoic acid)
SPR: Surface Plasmon Resonance
CD: Circular Dichroism
CM5: Carboxy Methyl 5
mM: milli molar
nm min^-1^: nano meter per minute
μl min^-1^: micro litre per minute
μM: micromolar
M: Molar
PBS: phosphate buffer saline
%: percentage
IC_50_: Inhibition concentration to inhibit 50% of enzyme
mgml^-1^: milligram per milliliter
min: minutes
s: second
Ms: millisecond
KD: equilibrium dissociation constant
deg cm^2^ dmol^-1^: degree centimeter square degree per molar
°C: degree Celsius
